# The QWERTY Effect: How typing shapes the meanings of words.

**DOI:** 10.3758/s13423-012-0229-7

**Published:** 2012-03-03

**Authors:** Kyle Jasmin, Daniel Casasanto

**Affiliations:** 1 Institute of Cognitive Neuroscience, University College London, London, UK; 2 Laboratory of Brain and Cognition, National Institute of Mental Health, Bethesda, MD USA; 3 Neurobiology of Language Department, MPI for Psycholinguistics, Nijmegen, The Netherlands; 4 Donders Institute for Brain, Cognition, & Behaviour, Nijmegen, The Netherlands; 5 Department of Psychology, The New School for Social Research, 80 Fifth Avenue, 7th Floor, New York, NY 10011 USA

**Keywords:** Motor action, Meaning, Orthography, Typing, QWERTY, Valence

## Abstract

The QWERTY keyboard mediates communication for millions of language users. Here, we investigated whether differences in the way words are typed correspond to differences in their meanings. Some words are spelled with more letters on the right side of the keyboard and others with more letters on the left. In three experiments, we tested whether asymmetries in the way people interact with keys on the right and left of the keyboard influence their evaluations of the emotional valence of the words. We found the predicted relationship between emotional valence and QWERTY key position across three languages (English, Spanish, and Dutch). Words with more right-side letters were rated as more positive in valence, on average, than words with more left-side letters: the *QWERTY effect*. This effect was strongest in new words coined after QWERTY was invented and was also found in pseudowords. Although these data are correlational, the discovery of a similar pattern across languages, which was strongest in neologisms, suggests that the QWERTY keyboard is shaping the meanings of words as people filter language through their fingers. Widespread typing introduces a new mechanism by which semantic changes in language can arise.

**Electronic supplementary material:**

The online version of this article (doi:10.3758/s13423-012-0229-7) contains supplementary material, which is available to authorized users.

For many people, language may be typed and read almost as much as it is spoken and heard. The phrase “talk to you later” (abbreviated *ttyl*) often means that conversational partners will continue “talking” with their fingers. When they do, they are likely to use the QWERTY keyboard. The QWERTY layout was invented in 1868 (Logan & Crump, 2011) and later sold to Remington to replace their alphabetical layout, which caused neighboring keys to jam during fast typing. QWERTY was designed to separate frequently used letter pairs to opposite sides of the keyboard, avoiding mechanical clashes. Additionally, QWERTY placed the letters in “T-y-p-e W-r-i-t-e-r” conveniently on the top row of keys, to help salesmen tap out what was, at one time, a brand name (David, 1985).

The QWERTY keyboard is now everywhere in our culture. Coffee shop chatter is being replaced by the sound of clicking keystrokes. Smart phones and laptops let people type messages virtually anywhere. Routinely, language is produced without speech. When linguists and psychologists talk about the *articulators* used in language production, they usually mean parts of the vocal tract. But increasingly, the articulators that produce our day-to-day language are the fingers.

The way words are articulated with the mouth is related to their meaning. Although many sound–meaning mappings are arbitrary (de Saussure, 1966), there are aspects of meaning that appear to be nonarbitrarily linked to the configuration of the vocal tract articulators used to produce them (Ohala, 1984). Here, we propose a link between the meanings of words and the action of the *manual* articulators used for typing them. Because patterns of articulation are not independent of meaning, typing may introduce a new mechanism by which semantic changes in language can arise.

Typing is a special kind of motor action. Performing motor actions fluently generally leads to positive feelings and evaluations (Oppenheimer, 2008; Ping, Dhillon, & Beilock, 2009). Therefore, if letters on one side of the keyboard can be typed more fluently than letters on the other side, motor fluency could mediate relationships between the locations of letters on the QWERTY keyboard and the valence of the words they compose (i.e., the positivity or negativity of their meanings).

The QWERTY keyboard is asymmetrical: There are more letters on the left of the midline than on the right. Therefore, striking one key among its neighbors should be more difficult on the left side of the keyboard than on the right side, due to greater response competition (Ridderinkhof, van den Wildenberg, Segalowitz, & Carter, 2004). This proposal is supported by reaction time data showing that when participants are presented with letters in isolation and are asked to press the corresponding keys, they are faster to type letters from the right side of the keyboard than from the left (Logan, 2003). Since this left–right asymmetry is built into the keyboard, it should affect skilled and unskilled typists alike.

If letters on the right of the keyboard are easier to type, this should lead to positive feelings when people type words composed of more right-side letters and negative feelings when they type words composed of more left-side letters. Associations between typing fluency and emotion could cause “right-side words” to acquire more positive valences and “left-side words” more negative valences. People who know how to type implicitly activate the positions of keys when they read words (Logan & Crump, 2011; Rieger, 2004). Therefore, typing experience could influence the valence of words that people read or speak even when people are not typing, as has been shown previously for evaluations of meaningless letter strings (e.g., Beilock & Holt, 2007; Van den Bergh, Vrana, & Eelen, 1990).

Here, we explored the relationship between QWERTY key position and word meaning in three experiments. In Experiment 1, we tested for associations between left–right key position and emotional valence in words from three QWERTY-using languages (English, Spanish, and Dutch). In Experiment 2, we tested whether words coined after QWERTY’s invention show a stronger association between key position and valence than do older words. In Experiment 3, we tested whether QWERTY key position affects the valence of pseudowords that have never been seen or typed.

## Experiment 1: Does QWERTY predict valence ratings for words across languages?

Experiment 1 tested for associations between the side of the QWERTY keyboard on which letters are located and the emotional valence of words that are spelled with these letters, in three languages.

### Method

#### Materials and procedure

We analyzed valence-normed words from three corpora (see Appendix A in the Supplementary online materials): the Affective Norms for English Words corpus (ANEW; Bradley & Lang, 1999), and two translation equivalents of ANEW in Spanish (SPANEW; Redondo, Fraga, Padrón, & Comesaña, 2007) and Dutch (DANEW).

ANEW consists of 1,034 words. Participants used a pencil to rate valence on a 9-point scale composed of five self-assessment manikins (SAMs), which ranged from a smiling figure at the positive end of the scale to a frowning figure at the negative end. Participants were told to mark one of the manikins or a space between two adjacent manikins (see Bradley & Lang, 1999). In SPANEW, translations of the ANEW words were rated by native Spanish speakers using a similar procedure (see Redondo et al., 2007).

DANEW was created for the present study, adapting Bradley and Lang’s (1999) procedure for computerized data collection (see Appendix B in the Supplementary online materials). The 1,034 ANEW words were translated into Dutch by a native speaker. Three of the English words translated to the same Dutch word. Removing these duplicates left 1,031 words in the sample. Each of the participants (*N* = 132 native Dutch speakers; 14 left-handers, 118 right-handers by self-report) saw 85 of the translated ANEW words intermixed with 74 words from an unrelated experiment, which served as fillers. Participants saw words one at a time and rated them for emotional valence on 9-point SAM scales (ratings for arousal, dominance, imageability, and concreteness were also collected; see Appendix B). Whereas ANEW and SPANEW participants used pencil and paper, DANEW participants responded by clicking one of nine radio buttons located beside the five manikins or between two manikins. In ANEW and SPANEW, the manikins were arranged from left to right. In DANEW, the manikins were arranged vertically on the screen, to avoid any unintended interactions between a left–right rating scale and the left–right positions of the letters that composed stimulus words.

### Results and discussion

For each word in the corpus, we computed the difference of the number of left-side letters (q, w, e, r, t, a, s, d, f, g, z, x, c, y, b) and right-side letters (y, u, i, o, p, h, j, k, l, n, m), a measure we call the *right-side advantage* [RSA = (# right-side letters) − (# left-side letters)]. Overall, there was a significant positive relationship between RSA and valence in ANEW, SPANEW, and DANEW combined, according to a linear regression with items (ANEW words and their translation equivalents) as a repeated random factor using SPSS’s GLM function [*b* = .044, Wald *χ* ^2^(1) = 5.34, *p* = .02; see Appendix C]. Words with more right-side letters were rated to be more positive, on average, than words with more left-side letters. We call this relationship *the QWERTY effect*.^1^

To determine whether the QWERTY effect differed across languages, *language* was added to the regression model as a fixed factor. The mean valence ratings differed between languages, producing a main effect of language [mean valence ratings: Dutch = 5.07, *SD* = 2.27; English = 5.15, *SD* = 1.99; Spanish = 4.74, *SD* = 2.14; Wald *χ* ^2^(2) = 101.09, *p* = .0001]. Importantly, however, language did not interact with RSA to predict valence [Wald *χ* ^2^(2) = 0.23, *p* = .89], and the effect of RSA on valence remained significant when the effect of language and the interaction of language with RSA were controlled [*b* = .043, Wald *χ* ^2^(1) = 5.19, *p* = .02].

Since there was no significant difference in the strength of the QWERTY effect across languages, an analysis of each separate language is neither required nor licensed. With that caveat, we note that the predicted relationship between RSA and valence was significant in English [*b* = .043, Wald *χ* ^2^(1) = 4.61, *p* = .03] and in Dutch [*b* = .051, Wald *χ* ^2^(1) = 5.81, *p* = .02], and a trend in the same positive direction was found in Spanish [*b* = .035, Wald *χ* ^2^(1) = 1.04, *p* = .31]. It would be inappropriate to interpret these patterns as differing between languages, given the lack of any statistical difference (Wald *χ* ^2^ < 1), which cannot be attributed to a lack of power (minimum *N* = 1,031 items).

A further analysis was conducted to control for possible effects of word length and for the frequency with which individual letters are used in each language (letter frequency).^2^ RSA remained a significant predictor of valence when word length, letter frequency, language, and their interactions were controlled [*b* = .057, Wald *χ* ^2^(1) = 6.95, *p* = .008].

A final set of analyses tested for effects of handedness in the DANEW raters (no information is available about the handedness of the raters for ANEW and SPANEW). People tend to implicitly associate their dominant hand side of space with positive ideas and their nondominant side with negative ones (Casasanto, 2009, 2011). For this reason, we added handedness to our model to test whether handedness would moderate the effect of RSA on valence. According to a mixed regression model using SPSS’s MIXED function, with subjects and items as repeated random factors, handedness did not interact with RSA to predict valence, *F*(1, 6077) = 0.16, *p* = .69, and RSA remained a significant predictor of valence when the effect of handedness and the interaction of handedness and RSA were controlled (*b* = .061), *F*(1, 1224) = 4.00, *p* = .05. Although the QWERTY effect did not differ significantly between right- and left-handers, we conducted an exploratory analysis to determine whether handedness influenced the direction of the correlation between RSA and valence. Right-handers showed a positive association of RSA with valence (*b* = .060), *F*(1, 1020) = 4.84, *p* = .03. Left-handers showed a trend in the same positive direction, which did not approach significance, likely due to the small number of left-handers (*b* = .022), *F*(1, 416) = 0.27, *p* = .61.

Overall, words with more right-side letters were rated to be more positive in meaning than words with more left-side letters, controlling for effects of language, word length, letter frequency, and handedness.

## Experiment 2: Does QWERTY influence new words more than old words?

In Experiment 2, we tested the QWERTY effect in a larger corpus of English words (AFINN; Nielsen, 2012), which included neologisms coined after the invention of QWERTY. We predicted that these new words should show a greater QWERTY effect than should older words, for three reasons. First, on average, the meaning of a newer word should be more malleable than that of an older word because it has a shorter history of use. Second, many of the new “words” in the AFINN corpus began as acronyms or abbreviations created by typists to facilitate the use of frequent expressions in social media (e.g., *LOL*, meaning *laugh out loud*). Words that are typed but rarely spoken should be particularly susceptible to biases introduced by the keyboard. Third, QWERTY may serve as a filter for the creation and use of new words. Words whose spatial locations are congruent with their valences (e.g., positive neologisms spelled with more letters on the right of the keyboard) might be remembered and used more often than incongruent words.

### Method

The AFINN corpus (Nielsen, 2012) consists of 2,477 English words that were rated for emotional valence on a 10-point scale (−5 to −1 and 1 to 5; see Appendix A). For consistency with our other experiments, we removed the unused zero point and renumbered the scale from 1 to 10. We identified 63 words in AFINN (including Internet acronyms) that were coined after the invention of QWERTY (see Appendix D). If a word’s date of origin was in question, we consulted www.etymology.com or www.urbandictionary.com.

### Results and discussion

RSA was calculated as in Experiment 1. Overall, there was a significant positive relationship between RSA and valence according to a linear regression [*b* = .037, Wald *χ* ^2^(1) = 9.15, *p* = .002; see Appendix C]. The effect of RSA on valence was significant in pre-QWERTY words, alone [*b* = .029, Wald *χ* ^2^(1) = 6.00, *p* = .014], but was much stronger in post-QWERTY words, alone [*b* = .363, Wald *χ* ^2^(1) = 9.00, *p* = .003], as indicated by the interaction of newness with RSA [Wald *χ* ^2^(1) = 17.75, *p* = .001]. Finally, as in Experiment 1, RSA remained a significant predictor of valence when word length, letter frequency, and their interaction were controlled [*b* = .06, Wald *χ* ^2^(1) = 19.06, *p* = .001].

## Experiment 3: Does QWERTY predict valence judgments for pseudowords?

Experiment 3 tested whether the QWERTY effect would be found for pseudowords, with no preexperimental meaning. This experiment addressed two questions raised by Experiments 1 and 2. First, could the QWERTY effect arise at a sublexical level (i.e., letters or clusters of letters)? Second, could the QWERTY effect be an artifact of lexical frequency? In principle, if words with higher RSAs also had higher frequencies, this could result in a spurious correlation between RSA and valence. Information about lexical frequency was not available for all of the words from Experiments 1 and 2, complicating an analysis to rule out possible frequency effects. In the present experiment, however, all items were novel and, therefore, had frequencies of zero.

### Method

#### Participants

Native English speakers (*N* = 800; 36 left-handers, 751 right-handers, 13 ambidextrous by self-report) were recruited via the Amazon Mechanical Turk Web site and participated online for payment.

#### Materials and procedure

A corpus of pronounceable, single-syllable English pseudowords was generated by crossing 46 consonant (or cluster) onsets and 18 consonant codas, for a total of 828 onset–coda combinations (consonant frames [CFs]). These CFs were crossed with four vowels (e.g., *pleek*, *plook*, *plake*, *ploke*) to make 3,312 pseudowords. CFs with actual words, or their homophones, were excluded, and four additional CFs were removed at random, leaving 1,600 pseudowords presented in a Latin square design (see Appendix A, Appendix E). Participants were instructed to read words in “an alien language” and to indicate how positive the meaning seemed on a 9-point scale by clicking a radio button. Each rated 20 words.

### Results and discussion

RSA was calculated as in Experiments 1 and 2. There was a significant positive relationship between RSA and valence, according to a mixed linear regression with subjects and items as repeated random factors (*b* = .027), *F*(1, 1584) = 10.85 *p* = .001 (see Appendix C), replicating the main results of Experiments 1 and 2. When handedness (for left- and right-handers) was added to the model, it did not interact with RSA to predict valence, *F*(1, 15053) = 0.03, *p* = .86. In further exploratory analyses, right-handers showed a positive association of RSA with valence (*b* = .029), *F*(1, 1574) = 11.76, *p* = .001. Left-handers showed a trend in the same positive direction, which did not approach significance (*b* = .025), *F*(1, 600) = 0.68, *p* = .41. As in Experiment 1, there was no effect of handedness on the association between RSA and valence.

Pseudowords with more right-side letters were judged to have more positive meanings in an alien language, suggesting that space–valence associations are stored or activated at the level of letters or combinations of letters. Importantly, the QWERTY effect in Experiment 3 cannot be explained by an unexpected relationship between lexical frequency and key position, since all items were novel and had frequencies of zero.^3^

## General discussion

In three experiments, we demonstrated a previously undocumented relationship between the meanings of words and the way they are typed: the QWERTY effect. On average, words spelled with more letters on the right of the keyboard were rated to be more positive in emotional valence than words spelled with more letters on the left. This was true even when raters were not typing. A similar relationship between word meaning and key position was found across three languages (English, Spanish, and Dutch). The QWERTY effect extended to pseudowords and was especially strong in neologisms coined after the invention of QWERTY, including abbreviations developed for texting. It appears that using QWERTY shapes the meanings of existing words and may also influence which new words and abbreviations get adopted into the lexicon and the “texticon” by encouraging the use of words and abbreviations whose emotional valences are congruent with their letters’ locations on the keyboard.

### Does typing fluency give rise to the QWERTY effect?

Since the present data are correlational, establishing the causal relationships underlying the QWERTY effect will require further research. We proposed that asymmetries in the distribution of letters over the left and right sides of the QWERTY keyboard should lead to more response competition among letters on the left of the keyboard and, therefore, to greater fluency in typing letters on the right. In turn, letters that are easier to type should come to carry more positive associations (and letters that are harder to type more negative associations) and should subtly influence the emotional valence of the words they compose.

This proposal is broadly consistent with previous research showing influences of typing fluency on preference judgments for meaningless letter strings (e.g., Beilock & Holt, 2007; Van den Bergh et al., 1990). However, previous studies have focused on different sources of typing fluency, such as finger repetition. For example, skilled typists prefer pairs of letters typed with different fingers (“f–j”) over pairs typed with the same finger during standard touch typing (“f–v”; Beilock & Holt, 2007). In exploratory analyses, we found no significant relationship between the number of finger repetitions in a word and its valence, nor was there any relationship between valence and the number of hand alternations used when typing a word—for any of the corpora we analyzed.

These other sources of typing fluency are orthogonal to the number of right-side and left-side letters in a word, and the effects we report here remain significant when both finger repetition and hand alternation are controlled. Further investigation would be needed to determine why our variable of interest, the RSA, predicted the valence of words reliably, whereas these other fluency-related variables did not. We note, however, that finger repetition (and to a lesser extent, hand alternation) should produce fluency effects only in skilled touch typists, who use the prescribed finger–key mappings. We have no information about the typing skill of the raters for the five corpora we analyzed, but we assume that it varied among raters. Importantly, if the QWERTY effect depends on asymmetries built into the keyboard per se, it should obtain for all frequent QWERTY users, regardless of their typing skill, typing style, or handedness.

### Alternative accounts of the QWERTY effect

The fact that we found no differences in the strength of the QWERTY effect across English, Spanish, and Dutch argues against two alternative explanations for this effect. First, if the effect had been found in only one language, it could have been due to accidents of sound symbolism (Ohala, 1984). In any single language, it could happen by chance that words with higher RSAs are more positive, due to sound–valence associations. But despite some commonalities, English, Dutch, and Spanish have different phonological systems and different letter-to-sound mappings. To maintain this skeptical alternative, it would be necessary to posit that RSA correlated with *different* letter-sound–valence mappings in each of these three languages, with strengths that did not differ across languages.

Second, since the inventor of QWERTY was an English speaker, if we had found the QWERTY effect in English alone, it would seem plausible that implicit space–valence mappings had shaped the QWERTY layout, and not vice versa. People implicitly associate positive ideas with their dominant side of space and negative ideas with their nondominant side, causing right-handers to place positive things on their right side and negative things on their left (Casasanto, 2009, 2011). There is about a 90% chance that the QWERTY inventor was right-handed, so implicit space–valence associations could have biased him to place letters that carried positive associations on the right of the keyboard and letters with negative associations on the left. Presumably, however, such implicit associations would be based on the peculiar roles these letters play in English words or sounds. The finding of similar QWERTY effects across languages suggests that, even if English-based letter-space–valence associations influenced QWERTY’s design, QWERTY has now “infected” typers of other languages with similar associations.

On a third alternative account, the QWERTY effect could result from general effects of manual motor fluency, which are not directly related to typing. The implicit associations between valence and left–right space described above (see Casasanto, 2011, for a review) are established through patterns of fluent and disfluent actions with one’s dominant and nondominant hands. These associations then exert broad influences on judgments, even for abstract entities that can never be seen or touched. In principle, the QWERTY effect could reflect the general preference for things on the right side of space found in the right-handed majority.

The finding that left-handers showed a trend to associate positive valence with letters on the *right*, rather than the left, argues against this possibility but does not rule it out entirely. Words meanings are normative. Variation in the way words are used is constrained by the community of language users, which is composed mostly of right-handers. The valence of words, therefore, may be shaped by fluency-based preferences in right-handers; left-handers’ implicit preferences are overruled. Such normative influences are evident in linguistic metaphors and idioms (e.g., all English speakers must agree that the correct answer is the “right” answer—even if they are left-handed). It remains an open question whether “good” is associated with “right” on the keyboard because “good” is associated with “right,” more generally, in a world dominated by right-handers.

## Conclusions

Although the relationship between words’ forms and meanings is largely arbitrary, aspects of how words are produced can shape their meanings. In the past, language was only spoken and was therefore subject only to constraints on hearing and speaking. Now language is frequently produced by the fingers, and for millions of people, it is filtered through QWERTY. As people develop new technologies for producing language, these technologies shape the language they were designed to produce.

The meanings of words in English, Dutch, and Spanish are related to the way people type them on the QWERTY keyboard. Words with more right-side letters are rated as more positive in emotional valence than are words with more left-side letters. The finding of the QWERTY effect in neologisms, and even in pseudowords, suggests that new coinages in language may show effects of how they are typed immediately. People responsible for naming new products, brands, and companies might do well to consider the potential advantages of consulting their keyboards and choosing the “right” name.

## Electronic supplementary material

Below is the link to the electronic supplementary material.

ESM 1 (PDF 514 kb)

## Footnotes

1 The phrase *QWERTY effect* is sometimes used informally in economics to describe a product that is highly successful despite being inferior to its competitors. The semantic QWERTY effect we report here is unrelated.

2 Information about frequency of letter use across languages was obtained from http://en.wikipedia.org/wiki/Letter_frequency, accessed April 10, 2011.

3 It is possible that when judging the valence of pseudowords, participants activated real English words that were phonologically or orthographically similar. However, this is merely a source of noise and is unlikely to account for the observed RSA effect, since letters that are phonological or orthographic neighbors are unlikely to be *typographic* neighbors on the QWERTY keyboard.
