# Supplementary material for: The QWERTY Effect: How typing shapes the meanings of words
Source: Psychon Bull Rev. 2012 Mar 3;19(3):499–504. doi: 10.3758/s13423-012-0229-7 (PMC3348452; doi:10.3758/s13423-012-0229-7)
Supplement: Supplementary file 1 — (PDF 514 kb) [file 13423_2012_229_MOESM1_ESM.pdf]

Online Supplemental Materials

Appendices:

A. Minima, maxima, means and standard deviations for Word Length, RHA, Count of Left Hand Letters, Count of Right Hand Letters, and Mean Letter Frequencies (per cent), for words in English ANEW, Spanish ANEW, Dutch ANEW, AFINN, and the Pseudoword corpus.
B. Complete list of words in the Dutch version of ANEW (DANEW), means and standard deviations of ratings for Arousal, Concreteness, Dominance, Imageability, and Valence.
C. Main results from Experiments 1-3 presented as weighted scatter plots of Right Side Advantage predicting Valence.
D. List of words from the AFINN corpus which post-dated the invention of the QWERTY keyboard.
E. Complete list of words in the Pseudoword corpus.

Appendix A: Minima, maxima, means and standard deviations for Word Length, RHA, Count of Left Hand Letters, Count of Right Hand Letters, and Mean Letter Frequencies (per cent), for words in English ANEW, Spanish ANEW, Dutch ANEW, AFINN, and the Pseudoword corpus:

English ANEW	Word Length	RHA	LH Letters	RH Letters	Mn Letter Freq
**Min**	3.0	-9.0	0.0	0.0	1.6
**Max**	13.0	7.0	10.0	8.0	10.1
**Mean**	6.2	-1.1	3.6	2.5	6.1
**SD**	1.8	2.5	1.7	1.4	1.2

Spanish ANEW	Word Length	RHA	LH Letters	RH Letters	Mn Letter Freq
**Min**	2.0	-14.0	0.0	0.0	3.7
**Max**	16.0	7.0	14.0	8.0	10.3

**Mean**	7.0	-1.2	4.1	2.9	7.3
**SD**	2.0	2.5	1.8	1.4	1.0

**Dutch ANEW**	**Word Length**	**RHA**	**LH Letters**	**RH Letters**	**Mn Letter Freq**
**Min**	2.0	-12.0	0.0	0.0	1.3
**Max**	20.0	6.0	14.0	11.0	14.7
**Mean**	7.1	-1.3	4.2	2.9	7.0
**SD**	2.6	2.7	2.1	1.6	1.9

**AFINN**	**Word Length**	**RHA**	**LH Letters**	**RH Letters**	**Mn Letter Freq**
**Min**	2.0	-11.0	0.0	0.0	1.8
**Max**	18.0	8.0	11.0	10.0	10.2
**Mean**	7.7	-1.7	4.7	3.0	6.2
**SD**	2.4	2.8	1.9	1.7	1.1

**Pseudowords**	**Word Length**	**RHA**	**LH Letters**	**RH Letters**	**Mn Letter Freq**
**Min**	4.0	-6.0	0.0	0.0	3.9
**Max**	7.0	5.0	6.0	5.0	14.5
**Mean**	5.3	-1.5	3.4	1.9	2.9
**SD**	0.7	2.2	1.2	1.1	1.9

Appendix B: Complete list of words in the Dutch version of ANEW (DANEW), means and standard deviations of ratings for Arousal, Concreteness, Dominance, Imageability, and Valence

Word	Arousal	(SD)	Concreteness	(SD)	Dominance	(SD)	Imageability	(SD)	Valence	(SD)
aangenaam	3.70	2.00	4.50	2.46	3.90	2.08	5.10	2.38	7.70	1.06
aanmoediging	7.00	1.10	6.50	2.59	7.67	0.82	7.17	1.17	7.83	0.98
aanstoot	5.00	2.76	4.00	2.53	6.50	0.84	3.00	3.03	3.33	0.82
aanstootgevend	5.10	2.42	4.60	2.55	5.70	2.58	5.00	2.58	3.30	1.70
aantrekkelijk	7.40	2.19	5.20	2.49	7.00	2.00	7.00	1.41	8.60	0.89
aanvaarding	6.29	1.60	3.71	1.60	5.57	2.15	5.00	2.71	5.14	2.34
aanval	7.11	1.45	6.67	1.94	7.22	1.39	6.78	1.86	3.44	1.81
aarde	3.60	1.95	7.20	2.95	5.20	2.68	7.20	2.17	6.60	1.14
aardig	2.70	1.57	5.30	1.16	4.00	1.83	6.50	1.35	7.70	1.16
abortus	7.25	1.67	7.75	1.28	7.13	1.46	6.75	1.67	3.13	1.96
absurd	6.00	1.07	5.63	2.77	7.13	1.36	5.00	2.78	4.00	1.51
achtbaan	8.29	1.11	7.57	1.40	6.14	2.12	8.57	0.79	8.14	0.90
activeren	6.43	1.99	6.71	1.25	5.57	2.70	6.57	2.07	7.00	1.41
afgemat	2.86	2.19	6.57	1.40	6.71	2.14	7.00	1.53	2.29	1.11
afgestudeerde	6.60	1.14	7.80	1.30	6.60	1.67	6.40	1.95	8.60	0.55
afgrijselijk	7.43	1.51	6.43	1.72	8.00	0.82	5.71	2.93	1.57	0.98
afstandelijk	3.83	0.98	4.50	1.87	3.67	1.51	5.50	1.52	3.00	0.63
afval	2.30	1.25	7.40	2.01	4.30	2.00	8.60	0.52	2.90	0.99
agressief	6.83	3.13	5.00	2.28	8.17	1.60	7.50	0.84	1.00	0.00
alcoholisch	4.00	1.00	7.20	1.48	5.20	1.30	6.60	2.07	3.60	1.52
alert	6.22	1.99	4.89	2.09	6.56	1.42	4.56	2.30	7.22	1.64
alimentatie	4.71	1.11	5.57	1.81	4.71	1.38	4.86	2.27	5.29	2.50
alleen	4.44	2.55	6.89	1.69	3.89	2.52	7.22	1.86	2.89	0.93
allergie	3.80	1.79	7.20	1.48	4.80	1.92	7.00	2.12	2.60	1.14
ambitie	7.50	1.07	4.75	2.60	7.50	1.41	6.50	2.51	7.63	1.41
ambulance	5.43	1.90	8.00	1.00	6.71	1.25	8.43	0.98	3.57	2.15
angst	7.86	1.68	6.00	3.32	7.86	1.21	7.00	2.65	2.00	1.00

animo	6.63	1.92	6.13	2.47	6.38	1.69	6.00	2.27	7.38	1.51
antwoord	5.20	1.10	7.60	1.14	6.60	1.67	6.40	2.88	5.20	1.48
apparaat	3.50	2.81	6.50	3.08	4.83	2.23	6.83	2.23	5.83	1.60
applaus	6.83	1.47	8.00	0.89	6.83	1.83	8.67	0.52	8.67	0.82
arm	4.86	1.77	6.14	2.34	3.57	1.51	7.57	1.90	4.00	1.91
armoede	3.90	2.77	7.00	2.11	6.60	1.84	7.30	2.11	1.70	0.95
aroma	5.20	1.30	6.80	0.84	5.60	1.14	4.60	2.70	6.60	1.34
arrogant	5.33	2.73	4.67	2.25	7.67	1.63	6.67	0.52	2.50	1.22
arts	4.43	2.07	7.86	1.21	6.14	2.34	7.86	2.04	7.00	1.15
astronaut	6.67	1.87	8.00	0.71	5.56	1.94	8.44	0.73	5.89	1.05
atletiek	6.00	1.00	8.60	0.55	4.00	2.00	8.40	0.89	5.80	1.92
atmosfeer	5.00	1.85	6.75	2.60	4.88	1.89	5.88	2.85	6.00	1.31
attent	4.44	1.51	5.44	2.19	5.44	1.67	4.67	2.06	7.78	1.20
auto	5.43	2.94	8.43	0.79	6.43	1.40	8.57	0.79	7.14	1.57
avontuur	8.14	1.46	7.29	2.14	7.86	1.68	7.14	2.73	8.00	1.00
baby	5.38	2.39	8.63	0.74	5.50	3.02	8.75	0.71	6.75	1.67
bad	4.40	3.21	9.00	0.00	4.60	2.88	9.00	0.00	7.60	1.34
badkamer	2.67	1.86	8.67	0.82	4.83	2.56	9.00	0.00	6.33	1.75
badkuip	4.17	1.33	8.83	0.41	5.17	2.14	9.00	0.00	7.17	1.47
bajes	3.10	2.33	8.60	0.70	5.70	2.41	8.60	0.84	2.40	1.35
bakken	2.80	2.05	6.40	3.21	3.20	2.68	7.00	2.55	5.80	1.30
bang	6.43	2.30	4.29	2.29	6.57	2.57	5.57	2.64	2.43	1.13
bar	5.86	0.69	7.71	0.76	5.29	0.95	8.14	0.90	6.86	0.38
bed	2.67	1.87	9.00	0.00	4.78	2.68	9.00	0.00	6.89	1.54
bedelaar	2.40	1.14	8.80	0.45	4.40	3.21	8.60	0.55	2.40	0.89
bedreiging	7.88	1.13	6.25	2.38	7.50	1.20	6.88	2.03	2.25	2.05
bedroefd	6.00	2.00	3.86	2.04	5.43	2.07	5.29	2.29	2.43	1.40
bedrog	4.86	2.67	5.00	2.89	6.00	1.91	5.71	1.89	1.57	0.53
beest	7.00	0.82	7.43	1.13	6.43	1.13	7.71	1.70	4.57	1.27
begraafplaats	3.88	2.47	8.75	0.46	7.00	1.93	8.75	0.46	1.88	0.99
begrafenis	5.60	3.13	8.20	0.45	6.60	2.61	8.20	0.84	1.00	0.00
behandelen	4.83	1.47	4.00	2.53	4.50	1.64	4.67	2.58	6.67	1.37
behendigheid	7.00	1.55	4.83	2.64	5.00	2.28	5.83	2.23	8.33	0.82

bekendheid	3.90	2.81	5.80	2.82	5.40	2.22	5.40	2.91	6.50	0.71
bekijken	3.40	1.67	6.20	1.30	2.60	1.34	5.60	1.34	6.20	1.64
bekwaam	4.80	3.03	5.60	1.34	4.40	2.19	4.20	1.48	7.40	1.14
belachelijk	4.57	2.76	5.43	1.99	6.14	2.41	5.43	2.07	2.57	1.40
belang	5.00	1.00	3.43	1.62	5.57	1.72	3.29	1.25	5.57	0.98
belast	5.33	1.87	5.22	1.56	6.44	1.24	5.22	1.72	2.44	0.88
beledigen	5.20	2.17	5.60	2.97	4.60	2.51	5.00	2.74	1.80	0.84
belediging	7.50	1.20	5.38	1.92	6.75	1.28	6.63	1.85	2.00	2.07
beleefdheid	4.43	2.76	5.86	2.12	5.00	1.15	5.14	1.86	7.43	1.40
belemmeren	5.71	2.06	6.86	2.12	5.71	2.81	6.86	2.73	2.43	0.98
beloning	6.86	2.73	6.71	1.70	6.43	2.64	6.71	1.50	8.43	0.79
beminnelijk	5.57	2.30	5.86	2.12	5.14	2.41	4.29	2.98	7.57	1.40
bemoeial	4.80	1.64	4.20	1.92	7.40	1.14	5.40	2.61	2.40	0.89
berg	4.33	3.08	8.83	0.41	6.00	2.76	8.67	0.82	5.33	1.37
beroemd	6.33	3.01	5.00	3.63	7.00	2.28	4.67	3.50	6.33	0.82
berouwvol	3.17	2.04	3.50	2.59	4.83	1.83	3.33	2.88	3.33	2.94
beschaamd	5.00	2.00	5.56	1.81	4.56	1.51	5.44	2.01	2.22	0.97
bescheiden	3.40	2.22	5.20	2.30	3.60	1.96	5.40	2.63	6.10	1.45
beschermd	5.00	1.58	5.80	0.84	5.40	1.95	4.80	2.17	7.20	0.84
beschut	3.86	1.21	4.00	1.63	3.86	1.35	3.71	1.89	5.71	0.95
bewolking	4.44	1.74	7.11	1.45	5.00	1.12	7.67	1.87	4.00	2.06
bewonderd	4.80	1.48	4.40	1.14	4.80	2.39	5.20	1.92	7.40	1.14
bezig	6.63	1.06	4.50	2.20	6.63	1.41	7.38	1.41	6.63	1.19
bezorgd	4.43	2.23	4.71	2.36	5.00	2.08	4.71	1.38	3.43	1.13
bijen	6.00	1.41	8.00	1.91	6.57	1.90	8.14	1.07	3.14	1.57
blaar	2.50	1.77	8.75	0.46	5.50	2.20	8.75	0.46	2.50	1.41
blasé	4.60	2.61	3.80	1.64	5.80	1.64	3.20	2.68	4.40	1.52
blauw	4.00	1.67	7.67	2.42	5.17	2.23	8.83	0.41	6.50	1.52
bliksem	7.67	1.51	6.67	2.73	6.67	1.86	6.33	3.08	4.00	2.19
blind	3.50	3.89	7.67	2.80	5.50	3.78	5.83	3.82	1.50	0.84
bloedbad	5.00	3.02	6.70	1.95	7.20	1.32	7.00	1.33	1.20	0.42
bloederig	6.00	2.71	6.29	1.80	6.00	2.45	6.71	2.21	2.29	0.95
bloedheet	4.80	2.17	7.00	1.22	6.40	1.52	7.60	1.14	3.00	1.87

bloem	4.57	2.37	8.29	0.76	4.86	1.21	8.29	1.11	8.14	0.90
bloesem	3.44	2.01	7.22	2.11	4.56	1.59	7.78	2.22	7.33	1.22
blond	4.29	3.30	6.86	1.86	4.57	2.44	7.71	1.38	6.00	1.41
bloot	7.60	1.67	8.80	0.45	5.60	2.79	8.80	0.45	6.40	1.67
blubber	5.00	1.85	7.38	1.92	5.25	2.31	8.00	0.76	3.63	1.92
boek	5.57	2.94	8.57	0.53	5.43	2.51	8.71	0.76	6.86	1.68
boeket	3.29	2.21	7.86	1.21	4.57	2.99	8.14	1.21	7.57	0.98
boerderij	4.43	2.51	9.00	0.00	4.57	2.23	9.00	0.00	6.00	1.29
boete	5.40	2.97	7.80	1.30	6.80	0.84	7.40	1.82	1.40	0.89
bol	3.00	2.19	7.50	1.76	3.50	2.17	8.17	0.98	5.67	1.03
bom	6.00	3.10	8.50	0.84	6.50	2.95	7.33	3.14	1.17	0.41
bont	4.20	0.84	7.60	1.52	4.60	2.19	7.20	2.05	4.00	1.41
boodschappen	3.70	1.25	7.90	0.88	3.80	1.55	8.40	0.70	6.30	1.16
boom	2.20	2.17	9.00	0.00	4.80	2.39	9.00	0.00	6.80	1.64
boomstam	3.71	1.11	7.14	1.95	4.00	1.41	8.71	0.49	6.14	1.46
boos	4.30	2.26	6.70	1.57	6.20	1.87	7.30	1.34	2.30	1.06
borst	5.56	1.88	7.78	1.64	6.22	1.09	8.00	1.22	6.33	1.00
boter	4.00	3.00	8.80	0.45	4.20	2.77	9.00	0.00	5.80	1.30
boxer	7.38	1.19	8.50	0.76	6.38	1.85	8.25	1.39	4.63	1.51
braaksel	3.00	2.65	7.14	1.21	5.00	2.58	8.14	0.69	1.86	0.90
brand	8.43	1.13	8.86	0.38	8.29	1.25	8.86	0.38	2.14	3.02
branden	6.14	2.04	8.43	0.79	7.00	2.08	8.14	1.21	3.71	1.25
brandkraan	4.60	2.97	8.60	0.55	4.20	2.77	8.60	0.89	3.80	1.30
brief	4.00	2.68	8.67	0.82	4.83	1.33	9.00	0.00	6.33	1.51
broer	6.00	2.19	9.00	0.00	7.00	1.41	9.00	0.00	7.33	1.63
bruid	5.50	2.55	7.80	1.23	5.10	1.45	8.60	0.70	7.60	1.26
bruiloft	4.80	2.68	8.80	0.45	5.20	1.48	8.60	0.55	7.00	1.22
buitenshuis	4.57	1.62	5.43	0.98	4.86	1.35	5.86	2.27	6.57	1.72
bus	3.33	1.50	8.44	1.67	4.11	1.96	8.44	1.67	5.67	1.66
cadeau	6.60	1.14	8.40	0.89	4.60	2.07	8.20	1.30	7.40	1.52
casino	6.13	2.75	7.38	2.72	6.13	2.10	8.38	0.74	4.25	2.12
cel	4.57	2.88	7.00	1.53	6.29	1.60	8.29	0.95	2.14	1.21
chantage	7.86	1.21	7.57	1.62	7.71	0.76	5.71	3.15	2.86	2.27

chaos	6.57	2.44	6.71	1.50	7.43	1.51	7.71	1.38	3.00	1.53
charme	6.60	1.82	4.20	1.30	6.00	2.55	5.20	2.28	7.80	0.84
chirurgie	6.00	2.97	7.00	1.79	7.00	1.79	7.83	1.17	5.33	2.42
chocolade	8.67	0.82	8.67	0.52	6.83	2.14	9.00	0.00	9.00	0.00
circus	5.40	2.37	7.60	1.84	5.10	1.66	8.00	2.21	6.10	2.56
cirkel	3.40	1.67	9.00	0.00	3.80	1.30	9.00	0.00	5.80	1.30
comedy	4.86	1.68	6.86	1.86	5.29	2.06	7.00	2.83	7.57	0.79
comfort	3.67	1.80	5.89	1.90	5.89	1.45	5.78	2.59	8.11	0.78
computer	3.80	2.17	8.40	1.34	5.00	2.45	8.00	1.73	6.40	1.67
concentreren	4.25	2.43	5.38	2.26	6.13	1.55	6.50	2.78	6.50	1.31
contanten	5.14	2.41	4.57	2.23	4.71	0.95	4.71	2.75	5.57	1.81
context	3.86	1.21	3.71	2.29	4.57	2.37	4.29	3.09	5.86	1.57
controle	5.38	2.56	6.38	1.51	8.00	1.41	5.63	2.33	5.63	2.20
corrupte	6.00	1.00	4.40	0.89	7.80	0.45	3.40	1.52	2.00	0.71
crash	7.00	2.90	7.00	2.28	7.67	2.80	7.50	1.64	1.67	0.82
criminaliteit	6.50	2.81	3.83	2.71	6.83	2.86	4.33	3.01	1.17	0.41
crimineel	6.00	2.26	5.80	2.35	6.30	2.16	6.70	2.00	2.10	1.20
crisis	4.40	2.61	6.20	1.30	6.00	1.41	5.60	2.61	1.80	0.84
cycloon	6.86	1.86	6.29	2.14	6.00	1.63	5.43	2.64	2.43	1.51
daglicht	4.33	1.94	7.67	1.58	5.56	2.19	8.11	1.05	7.67	1.41
dankbaar	3.80	1.64	5.00	2.92	4.00	2.45	4.80	2.39	7.20	1.92
danser	6.38	2.07	8.00	1.77	5.88	2.53	8.75	0.71	7.88	0.99
dapper	7.50	1.22	5.17	2.93	7.33	1.51	5.50	2.88	8.00	0.89
deel	3.29	1.89	4.71	2.63	3.71	1.50	3.43	1.62	4.86	0.38
demon	6.86	1.77	5.86	1.86	6.29	1.98	5.14	2.79	2.57	2.15
depressie	4.38	3.02	5.63	2.50	6.88	2.17	5.63	2.39	1.25	0.46
depressief	3.80	1.64	5.80	2.39	4.40	2.79	5.80	2.77	1.00	0.00
deserteur	4.60	2.97	6.80	1.64	5.20	0.84	3.20	3.27	3.00	2.00
detail	4.00	2.53	6.00	2.00	3.67	2.07	4.33	2.16	5.83	0.98
deugd	4.67	2.42	4.00	1.10	4.67	1.86	3.83	2.56	7.17	2.71
deuntje	4.50	1.72	6.70	1.57	3.40	1.58	7.40	1.96	7.80	1.03
deur	1.80	0.84	9.00	0.00	3.80	1.30	9.00	0.00	5.60	0.89
diamant	6.29	1.98	8.14	0.90	6.14	1.46	8.29	0.95	7.43	0.79

dief	7.33	1.50	8.00	1.32	6.33	1.73	8.00	1.32	1.78	0.83
diner	6.60	1.82	8.20	0.84	4.60	2.19	8.40	0.55	6.60	2.07
diploma	7.88	1.46	8.88	0.35	6.75	2.25	8.63	0.74	8.88	0.35
docent	4.14	1.57	7.43	0.98	5.43	1.27	7.86	0.90	5.57	0.79
dolk	7.43	1.27	8.43	0.98	7.71	1.25	8.43	1.13	2.86	2.34
dollar	4.86	2.19	8.86	0.38	6.71	1.11	8.71	0.76	7.00	1.63
donker	6.29	2.21	8.29	0.95	5.57	2.44	8.29	0.95	5.86	1.07
dood	6.20	3.27	6.60	3.58	6.60	3.58	5.60	3.85	1.80	1.79
doodsbang	6.00	3.24	5.80	2.86	6.80	1.79	7.00	1.22	1.40	0.55
doodskist	5.60	2.07	8.60	0.89	6.40	2.88	9.00	0.00	1.40	0.55
doorgang	3.50	2.35	6.50	1.52	4.83	2.40	6.00	2.83	6.17	0.98
doorn	5.50	1.64	8.17	2.04	6.00	2.37	8.17	2.04	2.50	1.52
dorp	3.60	1.96	7.20	1.03	3.10	1.60	8.20	0.92	6.30	1.57
drank	4.40	2.07	7.20	0.84	5.60	1.52	7.60	0.89	5.60	2.19
dromen	6.00	2.16	5.43	1.40	4.29	1.60	4.57	2.44	7.14	1.21
druk	6.14	2.54	6.43	1.81	7.43	1.51	6.86	1.68	4.43	1.40
duif	3.11	1.83	8.89	0.33	4.11	1.76	8.89	0.33	6.78	1.64
duiker	6.40	1.14	8.80	0.45	4.60	2.97	8.00	1.00	6.20	1.64
duivel	6.75	2.05	6.88	2.59	8.00	1.20	7.50	2.33	1.88	1.46
dwaas	4.17	2.40	4.67	1.21	4.00	2.10	5.33	2.42	3.00	1.26
echtgenoot	6.29	2.36	7.86	1.21	6.71	1.38	8.00	1.53	7.29	1.50
echtgenote	5.14	2.19	8.14	1.07	7.14	1.57	8.57	1.13	7.57	1.27
echtscheiding	5.29	3.15	8.57	0.79	7.86	1.46	7.43	2.70	1.43	0.79
eenheid	3.20	1.30	3.00	2.12	4.00	1.41	3.80	2.95	7.40	1.34
eenzaam	4.11	2.57	5.22	1.09	4.78	2.64	5.78	1.99	1.89	0.93
eenzaamheid	2.17	0.98	5.67	3.01	3.00	2.90	7.67	1.51	1.83	0.98
eer	5.67	2.66	4.50	3.27	8.00	0.89	4.33	3.08	8.17	1.33
eerbiedig	5.13	2.17	4.88	2.53	6.50	1.41	6.88	1.73	7.38	1.19
eerlijk	4.00	2.55	4.40	2.19	4.60	1.14	5.20	2.39	8.20	0.84
egoïstisch	6.00	1.80	5.56	2.01	6.56	1.94	5.33	2.50	2.11	0.93
ei	2.60	1.43	8.60	0.52	2.70	1.16	8.80	0.63	5.80	1.62
elegant	5.50	3.02	6.17	3.37	6.00	2.53	7.17	2.56	7.83	2.04
elleboog	2.40	1.67	9.00	0.00	4.40	2.19	9.00	0.00	5.60	1.34

ellende	6.43	1.90	4.57	2.23	5.71	1.80	5.14	2.41	1.29	0.49
energiek	7.43	1.13	5.43	2.37	6.71	1.50	5.71	2.14	8.00	1.00
engel	4.89	2.37	6.33	2.87	5.11	1.76	7.44	2.30	7.67	1.41
enkel	3.80	2.17	7.20	3.49	4.20	1.79	9.00	0.00	5.80	1.10
ernstig	4.00	2.19	4.67	2.73	6.17	1.33	5.83	2.71	2.33	1.51
erotisch	8.25	0.89	5.00	2.62	6.88	1.36	7.63	1.69	7.50	1.20
eten	6.14	2.54	7.00	1.29	6.71	1.50	8.00	1.15	7.43	1.40
excuus	4.71	2.75	6.29	2.36	5.57	2.37	5.00	2.58	4.86	2.12
extase	8.00	1.51	5.13	1.96	7.00	1.77	6.38	2.00	7.88	1.25
failliet	6.88	2.23	6.00	2.27	6.88	1.96	5.00	2.56	1.63	0.74
familie	7.00	1.41	8.40	0.55	6.60	2.70	8.80	0.45	8.80	0.45
fantasie	5.67	2.42	3.33	2.07	6.00	2.53	4.67	3.14	7.67	1.21
fascineren	7.67	1.21	4.83	2.40	6.17	2.48	6.17	2.14	8.33	1.03
fase	2.60	1.71	4.30	2.63	3.50	2.22	3.20	2.25	5.40	0.70
feestdag	4.20	1.92	7.40	1.14	4.00	2.45	7.00	2.83	7.60	1.52
feestelijk	6.57	2.07	5.71	1.80	5.29	2.21	6.86	1.57	8.14	0.90
feestje	7.57	0.98	6.43	1.99	5.43	2.07	6.29	2.69	7.86	1.07
fier	5.86	2.48	5.43	2.82	6.43	2.15	5.57	2.82	7.00	1.53
film	5.44	2.24	8.00	0.87	5.11	1.62	8.56	0.53	7.44	1.13
fles	3.40	2.61	9.00	0.00	3.80	2.77	9.00	0.00	6.60	2.19
flirt	8.25	1.16	4.63	3.11	6.13	1.36	8.00	0.93	7.75	1.67
foltering	4.14	2.85	6.29	2.14	5.29	2.69	3.57	1.90	2.00	1.83
fortuinlijk	7.00	1.29	6.43	2.44	7.00	1.29	6.43	2.94	7.43	1.13
fout	3.80	2.35	6.60	2.91	6.80	2.49	6.50	2.55	2.30	1.42
fraude	4.43	3.21	6.71	2.14	6.14	2.97	4.57	3.26	1.43	0.79
frigide	4.20	1.79	3.60	1.95	3.40	2.61	4.00	2.35	4.20	2.95
gang	2.50	1.76	7.50	1.52	3.33	2.07	8.00	2.00	5.17	0.41
gangreen	4.67	3.61	5.83	3.54	6.83	1.60	3.33	3.67	3.50	2.51
gazon	2.80	1.32	8.40	0.84	2.40	1.17	8.80	0.63	6.30	1.06
geïmponeerd	4.40	2.01	4.60	2.01	6.00	1.94	3.70	2.41	4.90	1.73
geïnspireerd	5.60	1.95	3.00	1.58	5.00	2.35	4.20	2.59	8.00	1.00
geallarmeerd	7.86	1.68	7.29	1.50	7.71	1.11	6.71	2.50	4.00	1.63
gebeurtenis	3.60	1.95	4.00	3.32	4.60	1.52	2.20	1.30	5.20	0.45

gebouw	3.57	1.81	7.43	1.72	4.57	1.51	8.14	0.90	4.86	0.69
gebroken	5.00	1.41	6.60	0.55	5.00	2.12	7.60	0.55	2.60	0.55
gebruik	4.56	2.13	4.56	2.24	5.78	1.30	3.67	1.80	5.89	1.17
gedachte	4.00	2.00	6.00	3.00	4.00	2.00	4.20	2.59	6.20	1.64
geel	6.00	2.88	8.38	0.92	6.13	1.73	8.75	0.46	7.13	1.46
geest	6.00	2.45	5.43	1.51	6.00	2.00	5.29	2.21	5.29	0.76
geesteszieke	5.14	2.73	6.86	1.95	5.71	2.63	5.86	2.79	2.14	1.46
gefluit	4.25	1.16	7.25	1.98	4.25	1.83	8.00	0.93	6.38	1.30
gefrustreerd	6.80	1.10	5.40	1.14	6.40	2.51	7.00	1.22	2.20	1.10
geheugen	3.83	3.25	6.83	1.94	5.33	2.42	4.67	2.58	6.83	1.60
gehoorzamen	4.83	2.64	5.67	1.86	6.50	1.87	5.00	1.90	6.17	1.60
gekweld	5.00	2.00	4.40	2.07	5.20	2.68	4.40	2.07	2.40	2.07
geld	6.57	2.51	8.43	0.79	6.86	1.95	8.57	0.79	7.71	1.11
geleerde	4.33	2.35	7.11	1.62	5.22	2.54	7.11	1.83	6.89	1.36
gelei	4.00	2.00	6.60	2.30	3.40	1.82	6.40	2.07	4.80	0.45
geliefd	7.50	1.05	3.67	2.16	6.50	1.76	5.00	2.61	8.33	0.82
gelukkig	7.25	1.58	6.25	1.75	7.50	1.41	7.50	1.60	8.75	0.71
gelukzaligheid	5.71	2.14	5.14	2.34	6.14	2.04	3.57	1.90	8.14	1.46
gemakkelijk	3.43	1.27	4.86	1.77	6.00	1.73	6.57	2.64	7.29	0.95
geneeskunde	4.75	3.06	7.25	1.91	6.38	1.92	6.75	1.98	7.00	1.93
genegenheid	4.80	2.39	2.80	1.10	4.00	2.65	3.20	1.92	7.60	1.67
genezen	7.00	1.90	4.33	1.51	5.83	1.17	5.33	2.25	8.17	0.98
genoegen	5.17	1.47	4.67	2.80	4.17	2.32	3.50	3.15	7.83	1.17
gerechtigheid	5.00	2.16	4.70	2.45	6.70	1.77	3.80	2.35	7.20	1.23
gereedschap	2.40	1.67	8.00	1.00	4.00	2.00	8.40	0.89	4.60	0.89
gereserveerd	4.00	1.63	4.57	2.15	4.57	1.40	4.57	2.70	3.86	1.21
geschenk	4.11	2.42	7.67	1.22	6.11	0.78	7.67	1.41	8.11	0.78
geschiedenis	4.60	1.82	6.60	1.67	4.00	1.73	2.80	1.10	5.60	1.14
geschokt	8.13	0.83	4.75	2.25	6.13	2.30	7.88	1.13	2.63	2.00
geschrokken	5.57	2.30	5.00	2.31	5.71	1.38	6.71	1.50	3.14	0.38
geslacht	4.25	1.58	7.50	1.77	6.13	1.64	7.13	2.47	5.25	0.46
geslachtsgemeenschap	8.60	0.55	8.20	1.10	7.00	1.58	8.60	0.89	8.40	0.89
gespannen	6.67	2.94	4.50	1.52	6.83	2.71	5.50	2.43	2.33	1.21

gespierd	5.20	1.93	7.50	0.97	7.10	0.99	8.60	0.52	6.60	1.43
getroost	5.33	2.25	4.33	2.80	5.17	1.47	6.33	2.73	7.00	2.28
gevaar	6.40	2.17	5.40	2.72	7.20	2.49	7.30	1.49	2.80	2.10
gevangenis	3.00	0.71	9.00	0.00	5.60	0.55	9.00	0.00	2.80	1.10
geweer	5.86	2.41	8.14	1.46	6.29	2.43	8.14	1.46	3.29	2.87
geweld	7.33	1.66	7.22	1.20	7.00	1.50	7.33	1.12	1.67	1.00
gewelddadige	5.40	2.51	7.80	0.84	4.60	1.52	7.80	1.30	2.20	2.17
geweldig	6.40	2.41	3.40	1.67	5.80	2.17	4.00	2.12	8.80	0.45
gewoonte	2.88	1.96	4.13	1.96	4.88	1.96	5.88	2.30	5.25	1.67
gezellig	5.40	1.51	5.60	1.65	5.80	1.23	6.70	2.26	8.20	1.14
gezicht	5.14	2.41	7.29	0.95	5.57	2.30	7.86	1.21	6.43	1.40
gezondheid	6.71	1.89	6.86	2.27	8.00	1.00	5.86	3.02	8.14	1.21
gif	5.29	2.63	7.43	2.94	5.86	2.73	7.00	3.06	1.43	0.79
giftig	5.00	2.00	7.40	1.67	5.40	2.97	7.00	2.12	2.00	1.22
gijzelaar	7.60	0.89	7.80	1.30	7.60	1.52	5.40	2.97	1.80	0.84
gil	6.33	2.07	6.33	2.34	6.67	2.58	7.83	1.47	3.33	1.86
glad	3.83	2.71	5.83	2.40	4.33	1.86	6.83	2.79	3.67	1.03
glamour	7.17	1.94	6.00	2.61	5.00	3.03	5.33	3.44	7.67	1.21
glas	3.10	2.02	8.10	1.91	4.20	2.35	8.00	2.54	4.90	0.32
gletsjer	3.00	1.22	8.00	1.73	5.20	1.30	8.60	0.55	6.00	1.41
gloeilamp	3.14	1.77	8.71	0.49	4.57	1.40	8.86	0.38	5.86	2.04
glorie	5.78	2.77	5.22	1.48	6.56	2.07	5.22	1.56	8.22	0.83
god	4.60	1.14	4.20	3.27	4.40	1.34	3.20	2.95	5.80	1.10
godslastering	7.00	1.93	5.75	3.06	6.25	2.38	6.00	2.33	3.00	1.77
goed	5.14	2.79	5.29	1.80	5.43	0.98	3.86	2.19	8.14	1.07
golfer	3.43	1.81	7.29	1.70	4.29	1.89	7.86	1.21	4.86	1.77
gordijnen	2.71	1.80	8.71	0.49	3.14	1.77	8.71	0.49	5.71	1.80
goud	5.13	2.85	8.50	0.93	5.75	2.82	8.75	0.71	7.38	1.51
graf	4.60	2.30	8.40	0.89	5.80	1.92	8.80	0.45	1.20	0.45
granaat	7.50	1.52	8.67	0.82	8.33	1.63	8.00	2.45	2.17	1.60
grap	7.50	1.64	7.67	2.16	7.50	1.52	7.00	2.28	8.00	1.26
gras	3.50	2.37	8.70	0.67	3.90	1.66	8.90	0.32	7.00	1.70
greep	5.60	0.55	4.40	2.51	5.80	1.92	3.80	2.05	4.60	0.89

grijns	5.14	2.12	6.71	1.60	5.57	1.99	7.00	1.83	8.00	1.15
groen	4.11	0.93	7.67	2.00	5.89	1.17	7.78	1.99	7.22	1.48
groet	4.80	2.77	7.00	1.58	3.80	2.68	6.00	2.65	7.40	1.14
groeten	4.88	1.64	6.38	2.33	4.38	1.69	8.00	1.41	7.88	0.83
grof	6.00	1.22	5.80	2.39	4.80	3.11	4.20	3.03	2.20	0.84
guillotine	5.57	3.64	7.29	1.70	6.00	2.65	7.43	2.88	1.43	1.13
gunst	6.14	0.90	5.71	1.38	6.57	1.51	5.71	2.63	6.71	1.60
gymnast	5.43	1.90	7.29	2.87	5.71	1.25	7.86	2.19	5.86	1.21
haai	6.40	0.89	8.40	1.34	7.20	0.84	9.00	0.00	3.00	0.00
haardroger	3.00	2.28	8.67	0.82	3.50	2.17	8.83	0.41	6.00	1.67
haarspeld	2.50	3.21	7.33	2.73	3.33	3.14	8.17	2.04	6.17	1.33
haat	5.80	3.16	6.30	3.50	7.30	2.31	6.20	2.44	1.30	0.67
hamburger	2.20	1.64	8.80	0.45	4.00	2.24	9.00	0.00	7.20	1.48
hamer	4.86	1.35	8.00	1.83	5.14	2.34	8.71	0.76	5.00	0.58
hand	4.11	1.76	8.33	1.41	5.22	1.20	8.56	1.33	5.89	1.45
handicap	4.40	2.97	6.40	2.88	4.40	3.44	5.40	2.19	2.00	1.22
hard	6.00	2.07	6.38	2.26	7.38	1.30	7.25	1.98	4.13	1.46
hardloper	5.57	1.72	7.71	1.11	5.71	1.11	8.43	1.13	5.86	1.86
hardvochtig	5.57	2.23	5.86	1.57	6.00	2.65	5.57	2.82	3.14	1.57
hart	5.14	2.73	8.14	1.57	6.29	2.87	8.29	1.50	6.14	0.90
haten	7.60	1.34	4.60	1.82	7.60	0.89	6.20	2.17	1.40	0.89
havik	4.67	3.14	9.00	0.00	4.50	1.76	7.67	3.27	5.50	0.84
hebzucht	5.83	2.48	5.33	2.73	7.67	1.51	6.17	3.31	1.83	0.98
heilige	3.30	1.83	6.00	2.31	4.70	2.11	6.40	2.80	5.30	1.95
hel	6.00	2.83	6.60	1.14	6.00	2.83	4.00	1.22	2.00	1.73
helder	4.14	2.04	4.57	2.37	4.86	2.19	5.43	2.23	7.29	1.70
hemel	5.00	1.73	4.33	2.78	5.89	2.03	4.33	3.50	7.67	1.00
herfst	5.40	2.30	8.00	1.00	5.40	2.97	8.60	0.55	6.40	2.41
herinneringen	5.50	2.07	5.50	2.93	6.13	2.47	7.75	1.39	7.13	1.46
heroïne	5.57	2.64	6.57	1.72	6.43	1.13	7.14	1.21	3.00	1.41
hoed	2.57	1.51	6.86	3.08	3.14	2.41	6.71	2.87	5.43	1.13
hoek	3.00	1.85	8.00	1.51	4.50	2.33	8.25	1.49	5.13	0.35
hoer	5.00	3.67	6.40	2.79	5.20	3.90	8.00	1.00	1.60	0.89

hond	5.17	2.99	8.83	0.41	5.33	2.94	9.00	0.00	4.83	2.71
hondsdolheid	3.50	2.81	4.67	3.27	4.83	2.56	5.00	3.79	1.50	0.84
hongerig	5.22	1.39	6.33	1.22	6.00	1.66	6.00	1.94	2.78	0.83
honing	3.00	1.83	7.70	1.83	3.50	1.84	8.70	0.48	7.00	1.15
hoofdpijn	3.60	2.41	9.00	0.00	4.20	2.05	8.60	0.89	1.80	0.45
hooi	3.29	1.38	8.29	1.11	4.00	2.24	8.14	1.07	5.57	1.13
hoopvol	6.33	2.12	5.00	1.80	6.44	1.51	4.44	1.94	7.33	2.29
hopen	6.60	2.51	5.20	2.17	6.00	3.32	4.80	2.68	7.40	1.14
hotel	4.88	2.03	8.38	1.06	5.63	2.45	8.00	1.77	7.13	0.35
huis	4.29	2.21	8.57	0.53	6.29	1.98	8.86	0.38	7.29	1.25
huisdier	5.14	1.68	8.43	0.98	4.71	1.70	8.14	1.57	7.57	1.81
huivering	4.38	2.00	5.50	1.60	5.88	1.46	6.50	1.07	2.50	0.76
hulpeloos	4.00	2.78	6.13	1.46	6.13	2.17	6.38	2.00	1.88	0.99
humeurig	2.50	2.35	5.00	1.26	6.67	1.97	6.83	2.04	2.00	0.63
humor	7.40	1.14	4.80	1.48	6.00	1.87	6.60	2.79	8.20	1.10
idee	6.17	2.14	5.00	2.28	6.17	1.33	4.33	3.08	7.67	0.82
identiteit	5.00	2.61	5.17	3.06	5.00	1.41	4.00	3.16	6.83	1.83
idioot	6.60	0.55	5.60	1.14	5.60	1.82	5.80	1.48	2.40	0.89
idool	4.60	2.22	7.50	2.12	5.00	1.94	6.40	2.27	5.80	2.44
ijdelheid	3.80	1.64	4.40	2.41	6.00	1.22	5.80	2.28	4.20	1.92
ijzer	4.43	1.51	7.71	1.11	4.86	2.19	7.14	1.77	4.57	1.27
immoreel	6.00	2.08	3.86	2.48	5.86	2.85	3.43	2.51	2.86	1.68
impotent	4.78	2.86	6.11	2.32	5.00	2.40	5.22	2.11	2.22	1.64
inbeelden	4.20	1.30	5.80	2.59	3.20	1.79	4.40	2.70	6.00	1.73
indringer	7.50	1.07	7.13	2.23	7.50	1.20	7.75	1.04	2.63	2.33
industrie	2.71	1.50	6.71	1.50	5.14	2.12	7.14	1.35	3.57	1.40
infectie	7.29	1.25	8.00	0.82	7.71	1.38	7.71	1.38	2.00	1.41
inferieur	2.20	1.30	3.20	2.68	3.80	2.28	3.20	1.30	3.80	1.64
inhoud	5.00	1.85	5.25	1.67	5.00	1.77	4.88	1.81	5.50	0.76
inkt	3.00	2.35	8.60	0.89	3.40	2.61	8.60	0.55	5.00	0.71
insect	3.50	1.52	8.17	1.60	4.00	2.10	8.50	0.84	3.50	1.64
inspireren	8.00	0.89	5.00	3.10	6.33	3.01	6.17	2.79	8.83	0.41
intellect	4.10	2.02	5.20	2.30	5.70	2.54	4.20	2.39	7.20	1.32

intiem	7.50	2.00	5.63	2.56	6.13	1.89	7.38	2.33	8.13	0.83
investeren	4.40	1.95	7.00	1.58	4.40	2.19	4.40	2.30	6.00	1.41
inwoner	4.00	1.29	6.14	1.68	4.29	1.98	5.43	2.23	5.00	1.00
irriteren	6.00	2.24	5.11	1.76	5.89	0.93	5.67	2.12	3.00	0.87
item	3.20	2.05	8.00	1.00	4.00	2.00	5.20	3.27	5.00	1.41
jacht	6.88	1.89	5.88	2.90	6.38	2.62	7.75	1.28	3.38	2.20
jaloezie	4.43	1.51	5.57	2.23	6.00	2.16	4.14	2.12	2.43	0.79
jammer	4.86	2.27	4.71	2.29	4.43	2.64	3.57	2.44	2.86	1.68
jarretel	4.14	3.98	7.29	3.15	4.43	2.94	7.00	3.06	6.14	1.95
jeugd	6.40	1.95	5.80	1.79	5.00	1.87	6.60	2.30	7.00	1.22
jong	4.90	1.97	6.20	2.49	5.40	2.01	7.00	1.70	7.30	0.82
jongen	5.83	2.40	8.33	1.21	5.67	2.34	8.67	0.52	6.33	0.82
jurk	7.83	1.60	8.50	0.84	5.83	2.04	8.83	0.41	8.33	1.21
juweel	5.60	1.65	7.40	2.07	5.50	1.51	7.90	1.37	7.30	1.25
kabinet	2.60	1.14	8.00	1.00	6.20	0.84	7.00	3.08	5.40	0.89
kachel	4.14	1.07	8.57	0.53	4.14	1.46	8.71	0.49	7.14	1.95
kakkerlak	5.89	1.54	8.44	1.01	5.56	1.42	8.11	1.27	3.33	1.22
kalmeren	4.00	2.12	5.80	2.86	4.00	2.45	7.20	1.79	5.40	1.67
kampioen	7.38	2.00	7.00	2.07	7.88	1.13	7.75	0.89	8.25	1.04
kanker	3.57	3.78	7.29	1.50	7.57	2.15	7.14	1.77	1.14	0.38
kanon	6.43	2.30	8.29	1.11	6.71	1.98	8.71	0.76	3.43	1.90
kans	6.57	2.37	6.14	1.57	6.71	1.50	5.57	2.23	7.29	0.95
kantoor	3.40	3.36	8.40	0.89	5.00	2.92	8.80	0.45	5.80	1.92
karkas	4.33	3.27	5.67	2.88	5.33	2.42	5.83	3.19	3.83	2.23
kat	6.17	1.94	8.67	0.52	6.00	2.76	8.50	1.22	7.83	1.60
katje	3.40	1.96	8.50	0.85	2.90	1.91	8.50	1.08	6.70	2.16
kelder	2.60	1.52	9.00	0.00	4.40	2.41	9.00	0.00	4.60	1.82
kennis	4.43	1.40	4.43	1.13	6.14	1.68	4.71	2.36	6.43	1.13
kerk	3.89	2.03	8.00	1.12	5.00	2.40	8.44	1.13	4.89	1.27
kerosine	5.20	1.92	8.60	0.55	4.00	2.45	5.40	2.51	3.80	1.64
kerstmis	6.50	1.93	7.50	1.77	7.63	0.92	8.63	0.52	8.13	0.99
ketchup	5.00	2.77	7.86	1.46	4.57	1.27	8.43	0.98	5.71	2.36
keuken	4.43	2.37	7.71	1.60	3.43	2.23	7.14	2.48	6.43	1.62

kids	3.88	2.36	7.00	2.20	4.88	2.47	8.25	1.16	6.63	2.50
kiespijn	5.40	2.97	7.80	1.30	6.60	2.30	6.60	2.70	1.40	0.89
kikker	2.67	1.97	9.00	0.00	3.50	1.97	9.00	0.00	5.33	1.03
killer	6.50	2.51	6.00	3.35	8.33	1.21	5.83	2.71	2.33	1.51
kin	2.60	2.12	8.80	0.63	3.20	1.62	8.80	0.63	5.10	0.88
kind	3.00	1.87	8.60	0.55	5.80	0.84	8.60	0.55	7.00	1.87
klap	6.86	1.21	6.14	2.79	5.86	2.73	7.14	2.12	3.43	1.40
kleding	4.78	1.92	7.56	2.46	6.00	1.66	8.56	0.73	6.78	1.30
kledingstuk	5.40	1.14	8.40	1.34	4.40	2.41	8.60	0.89	6.20	1.79
kleur	4.88	2.03	7.63	1.85	6.63	1.85	7.00	2.67	7.50	1.77
klif	5.43	2.07	6.43	1.90	5.71	1.38	7.57	0.98	4.86	1.57
klok	4.29	2.75	8.29	1.50	4.71	2.75	7.71	1.70	5.14	1.21
klont	3.71	2.75	8.29	0.95	4.00	2.00	8.57	0.79	3.43	2.15
kluis	2.80	1.92	8.40	1.34	6.20	3.11	9.00	0.00	4.80	1.10
knap	6.00	2.00	4.29	2.21	5.86	1.95	5.43	3.15	7.43	1.40
knijpen	5.00	2.76	7.50	2.26	5.50	2.51	8.67	0.52	3.17	0.41
knipoog	7.67	1.51	8.50	1.22	5.67	2.16	8.83	0.41	8.17	0.98
knoop	2.90	2.33	7.70	2.26	4.00	2.62	8.90	0.32	4.40	1.07
knuffelen	5.40	2.51	7.60	1.34	5.60	2.51	9.00	0.00	8.60	0.89
koe	4.43	1.51	7.71	1.60	5.14	0.90	8.14	1.46	6.57	1.81
kogel	7.33	1.32	8.33	0.71	7.56	0.88	8.00	1.32	2.56	1.42
koken	4.00	2.00	8.00	1.41	5.40	0.89	8.80	0.45	6.00	1.73
kolom	3.25	2.25	7.00	2.27	2.63	1.77	7.75	1.28	5.25	0.71
kolossaal	5.29	1.80	5.00	1.41	7.14	1.57	6.14	1.21	5.43	0.53
kom	2.57	1.40	7.57	2.15	2.86	2.54	7.57	2.15	5.29	0.76
konijn	3.25	1.67	8.50	1.41	3.63	1.92	8.50	1.41	6.50	1.69
konijntje	3.60	1.95	8.60	0.89	2.60	1.52	9.00	0.00	8.00	1.00
koning	4.17	3.25	8.67	0.82	7.83	1.83	8.33	1.63	7.00	1.41
koningin	3.50	2.07	8.83	0.41	6.83	2.79	8.50	0.84	7.33	1.03
koord	2.60	1.43	8.10	1.10	2.90	1.45	8.40	0.84	5.00	0.82
koorts	5.60	2.19	9.00	0.00	5.80	2.28	8.40	0.89	2.40	1.52
koplamp	4.43	1.62	7.29	2.14	5.43	1.13	8.43	0.79	5.71	1.50
koud	4.00	2.24	5.80	1.30	4.80	1.79	7.20	2.95	2.80	1.10

krachtig	6.29	2.06	3.43	2.30	7.14	1.57	4.00	3.21	6.71	0.95
krankzinnig	4.57	2.88	4.71	2.69	5.43	2.44	3.86	1.86	2.43	1.40
kroon	4.33	2.24	8.11	1.05	6.00	2.00	8.11	1.27	6.67	1.22
kruik	3.80	2.95	8.80	0.45	4.40	2.19	8.80	0.45	5.60	1.34
kruisigen	6.88	1.46	6.63	2.62	6.00	1.69	7.25	2.71	3.00	2.56
kruk	3.14	1.57	6.71	2.36	3.71	1.80	6.86	2.34	5.14	2.04
kunst	4.86	2.85	5.86	3.02	5.86	3.08	4.86	2.97	6.71	1.80
kurk	3.63	1.77	8.25	1.49	3.88	1.96	8.25	1.49	5.13	0.35
kus	8.20	0.84	8.40	0.89	7.40	1.14	9.00	0.00	9.00	0.00
kussen	5.33	2.88	7.67	2.34	5.00	2.61	8.50	0.84	7.00	1.67
kust	7.83	1.83	8.17	2.04	6.50	2.07	8.17	2.04	8.33	0.82
kwaadwilligheid	4.90	2.51	4.40	2.27	5.80	2.62	4.80	2.62	1.80	0.92
kwaal	2.60	2.07	6.60	1.82	3.60	2.41	3.80	3.56	2.00	1.22
kwaliteit	4.71	1.50	4.29	1.11	6.29	1.50	3.71	1.70	7.14	1.21
kwart	3.22	1.86	7.00	2.40	2.89	1.45	5.67	3.04	4.89	0.33
kwekerij	3.60	2.41	8.20	0.45	4.20	1.79	8.40	0.55	5.60	1.95
kwelling	7.00	1.20	4.50	2.45	7.00	0.76	7.25	1.83	2.38	2.39
kwetsen	5.00	2.77	4.57	2.70	6.14	2.04	3.86	2.54	2.14	1.07
laan	3.86	2.27	7.00	2.24	3.57	2.57	6.43	2.99	5.14	0.90
lachen	7.38	1.69	8.38	0.92	6.50	1.93	8.75	0.71	8.75	0.71
lafaard	2.80	1.64	4.60	2.07	4.00	2.92	3.80	1.79	2.40	0.89
lam	4.50	2.66	6.50	2.35	4.00	2.83	6.33	2.66	5.33	2.88
lamp	3.67	2.25	8.33	1.63	5.33	1.86	8.83	0.41	7.33	1.03
langzaam	2.88	1.89	6.38	2.45	4.75	2.12	7.88	1.13	3.63	1.06
lantaarn	3.00	1.89	8.70	0.67	3.50	2.17	8.90	0.32	6.30	1.06
lawaaierig	5.40	1.34	6.00	1.73	6.20	2.17	7.60	0.89	3.40	1.82
lawine	6.86	1.57	7.86	0.90	6.57	1.40	7.71	1.38	2.86	0.69
leed	5.56	2.40	6.22	1.20	5.89	1.54	6.11	1.96	2.00	1.12
leeuw	7.40	1.52	9.00	0.00	5.60	2.79	9.00	0.00	6.60	1.52
legende	6.38	1.19	5.25	2.60	6.13	1.73	5.88	2.17	6.88	1.64
leger	5.71	2.14	7.14	1.68	7.14	1.57	7.29	2.93	3.86	1.07
leider	5.71	1.50	8.00	1.15	7.71	0.76	7.71	1.60	7.43	1.13
lelijk	3.40	2.51	5.20	3.35	4.40	3.51	7.00	3.08	1.40	0.55

lenig	5.43	1.62	7.57	2.57	5.43	1.99	8.29	0.95	5.57	1.40
lente	6.00	1.73	7.80	0.84	5.40	1.67	7.80	1.10	8.40	0.89
lepra	4.33	3.72	6.67	1.63	5.17	2.99	4.17	2.86	1.67	1.63
leren	5.67	2.07	6.17	2.32	6.50	1.05	6.83	2.64	7.00	1.55
lesbisch	4.70	2.67	7.20	2.04	6.00	2.36	7.20	1.62	5.10	0.99
leugen	3.40	1.82	6.00	2.24	5.40	1.82	4.60	1.95	2.00	1.00
leuk	7.38	1.41	5.75	2.25	6.63	1.69	7.25	1.98	8.38	0.74
leven	7.14	1.21	5.29	3.09	7.29	1.38	5.00	3.21	8.14	0.90
levend	6.56	2.40	7.00	2.40	7.22	1.64	7.67	2.24	8.22	0.97
levendig	4.60	2.19	3.80	1.48	4.00	2.12	5.00	0.71	8.00	0.71
lichaam	4.60	2.07	8.20	1.79	3.80	2.17	7.80	2.17	6.20	1.79
lied	6.75	1.39	7.63	2.00	6.00	1.51	7.25	2.31	7.63	1.06
liefde	7.71	1.70	5.71	2.93	8.14	1.21	6.00	2.52	8.71	0.76
liefhebben	7.29	1.70	6.86	2.19	7.86	0.90	7.43	2.30	9.00	0.00
liefje	7.71	1.11	5.29	2.69	5.86	2.67	6.57	2.51	8.29	0.95
lift	2.60	1.52	8.40	1.34	3.40	1.82	9.00	0.00	5.60	0.89
lijk	3.67	3.01	8.00	1.55	4.83	2.71	8.17	1.17	1.00	0.00
lijkenhuis	3.50	3.89	7.50	3.21	6.33	3.01	6.00	3.95	1.83	1.33
litteken	4.40	2.63	8.30	0.82	5.30	1.77	8.50	0.71	3.00	1.63
lol	4.60	2.30	7.00	0.71	4.20	1.64	5.60	1.14	8.40	0.89
loser	4.14	1.77	4.14	1.35	3.57	1.99	4.29	2.43	2.86	0.90
loterij	6.11	2.09	7.56	0.73	5.22	1.48	7.33	1.87	5.78	1.64
loyaal	4.90	1.79	5.50	2.92	6.10	1.52	4.50	1.72	7.70	1.16
lucht	5.80	3.35	9.00	0.00	5.00	2.83	8.60	0.89	7.00	2.00
lui	3.40	2.30	6.60	0.55	3.40	2.07	6.20	0.84	4.00	1.22
luis	6.38	2.72	8.25	1.16	5.75	2.31	8.13	1.25	2.13	0.99
luizen	3.57	3.15	7.71	1.60	5.29	2.93	7.14	1.57	1.86	1.57
lukraak	4.71	1.70	3.00	1.15	5.00	1.63	3.14	2.48	4.29	2.06
lust	8.00	1.00	6.00	2.58	7.57	1.27	7.00	2.31	7.29	0.95
lusteloos	3.80	2.49	5.80	2.57	4.60	2.76	6.00	2.58	2.20	1.62
luxe	5.43	2.23	5.43	2.15	6.43	1.51	6.57	1.40	7.71	0.95
maag	3.25	1.98	7.63	1.77	4.75	1.98	8.13	1.36	5.00	0.53
maagd	4.00	1.41	7.40	1.14	5.00	2.92	6.80	2.39	5.20	0.45

maagzweer	4.67	3.39	7.67	1.51	4.83	3.25	6.50	2.35	1.33	0.82
maanden	4.50	1.52	6.83	2.99	5.17	2.56	3.33	2.34	5.83	0.98
machine	3.30	2.26	7.40	1.71	4.70	2.71	7.50	1.72	4.60	1.65
macht	4.60	2.07	7.00	0.71	7.20	1.79	4.80	1.64	4.20	1.10
machtig	7.44	1.24	5.56	1.74	7.56	1.88	6.11	1.90	6.33	1.66
made	5.43	2.23	6.43	2.51	4.71	2.50	6.29	3.04	2.86	1.95
magisch	7.00	1.53	4.29	2.06	6.43	1.40	5.71	1.60	7.57	1.40
malaria	6.56	1.94	6.11	3.02	5.44	1.42	3.67	1.80	1.56	0.88
man	4.40	3.13	8.40	0.89	4.80	2.49	8.60	0.55	5.40	2.97
mand	2.88	1.89	8.50	1.07	3.38	1.85	8.63	0.74	5.00	0.00
maniak	5.29	2.29	6.00	1.63	6.43	1.51	5.71	1.11	2.71	1.25
manier	4.71	0.95	4.29	1.38	4.86	1.07	2.86	1.57	5.57	1.13
markt	4.86	2.48	7.71	2.21	5.29	1.38	7.71	2.21	6.29	1.50
masturberen	8.40	0.89	4.60	2.88	7.00	0.71	6.80	1.64	7.80	0.84
materiaal	2.83	2.23	3.83	2.40	4.67	2.34	5.33	2.25	5.33	0.52
mazelen	3.33	3.14	6.67	1.63	5.67	1.63	6.33	2.94	1.83	1.60
meeldauw	2.40	1.35	4.20	2.97	3.50	1.35	2.80	2.20	4.70	0.95
meer	4.00	1.87	2.80	3.49	5.80	1.92	3.40	3.58	4.80	1.10
meesterlijk	7.00	1.79	3.83	2.14	7.67	1.51	4.17	2.32	8.50	0.84
meisje	4.57	1.90	8.43	0.53	5.00	2.24	8.43	1.13	7.71	1.38
melk	2.33	1.50	8.78	0.44	4.22	1.09	8.89	0.33	6.89	1.17
melodie	5.60	0.55	7.00	2.45	5.00	2.45	7.60	2.07	7.00	1.87
mening	5.50	2.27	4.38	2.39	6.25	2.05	5.75	2.82	6.25	1.28
mensen	5.29	2.50	6.86	2.04	6.43	2.30	7.71	1.98	7.14	1.68
menslievend	5.00	2.24	6.71	1.89	5.14	2.54	6.57	2.64	8.43	0.79
mes	4.86	2.19	8.43	0.98	6.86	1.35	9.00	0.00	4.00	1.53
mest	2.60	2.19	8.00	1.00	3.40	2.61	8.20	0.84	3.40	0.55
metaal	3.17	2.56	7.67	1.97	5.83	0.98	8.67	0.52	5.17	0.41
methode	4.17	1.83	3.67	1.03	3.67	2.66	3.17	1.47	5.67	1.03
mild	4.14	2.34	4.86	1.86	3.86	2.04	5.43	2.64	6.29	1.38
miljonair	5.90	1.60	7.50	0.97	5.70	1.70	6.30	2.75	7.20	1.23
minachtend	5.57	1.62	5.71	1.98	5.57	2.30	5.86	3.24	1.71	0.95
minachting	4.20	2.17	4.60	2.51	3.60	2.70	3.20	2.28	1.60	0.89

mirakel	6.00	2.71	3.57	2.07	6.00	2.08	3.43	2.70	6.43	2.15
misbruik	6.56	1.94	5.33	2.06	6.56	1.81	4.89	2.62	1.78	0.83
mislukking	4.60	1.95	5.60	2.07	3.80	2.59	5.00	2.12	2.20	1.64
misvormd	5.88	1.81	6.00	2.62	6.50	1.69	7.63	1.60	1.88	0.83
mobiliteit	4.29	2.56	5.29	1.38	5.14	1.57	5.43	1.13	6.43	1.27
modder	4.14	2.19	7.43	1.81	4.86	1.86	7.86	1.68	3.57	0.79
modderig	4.86	1.77	8.29	1.25	5.29	2.50	8.29	1.25	3.29	1.80
moeder	5.20	0.45	8.60	0.55	5.00	2.74	8.60	0.55	8.40	0.89
moedig	6.80	2.17	6.40	2.70	6.20	3.27	6.40	2.41	8.20	0.84
moeras	3.33	2.07	8.00	1.26	4.67	2.25	7.67	2.34	4.33	0.52
moment	3.67	2.16	5.17	1.47	4.33	1.86	5.17	2.99	6.00	0.63
moordenaar	6.60	3.17	7.70	2.50	7.20	2.44	7.30	2.75	1.30	0.48
morbide	5.40	2.70	2.60	1.67	5.80	3.11	1.60	0.89	3.20	1.79
moreel	5.14	2.19	3.71	2.63	5.29	1.89	3.00	2.08	5.86	1.77
morgenstond	3.78	1.79	4.00	1.80	4.00	1.32	3.67	2.40	6.00	1.58
motor	6.40	1.34	8.00	2.24	4.00	2.00	8.20	1.79	6.60	1.82
muffin	5.00	2.20	8.38	1.06	5.13	2.59	8.63	0.74	7.88	1.13
mug	3.57	2.23	8.29	1.11	6.43	1.72	8.43	1.13	2.00	1.00
munt	3.57	2.44	8.29	0.95	3.86	2.61	8.43	1.13	6.86	1.21
museum	4.71	2.14	8.71	0.49	3.71	2.14	9.00	0.00	6.57	1.90
muziek	7.60	0.89	6.20	2.95	5.60	2.97	7.80	2.17	9.00	0.00
mystiek	6.00	2.20	3.00	1.51	4.75	1.39	4.50	2.33	6.25	1.28
naakt	7.33	0.52	8.17	1.33	6.17	2.32	8.50	0.84	5.67	1.21
naald	4.83	2.79	8.33	1.63	5.67	0.52	8.33	1.63	3.83	1.17
naam	3.40	2.27	7.00	2.00	4.60	1.90	6.30	3.02	6.60	1.17
nachtmerrie	3.80	1.92	8.20	0.84	5.00	2.74	7.20	3.03	2.20	1.30
natuur	5.86	1.46	5.71	2.14	4.86	2.48	6.57	2.76	7.86	0.90
natuurlijke	3.78	1.56	4.33	1.50	4.78	1.56	4.33	1.00	6.89	0.93
nectar	4.20	1.10	7.80	2.17	3.60	2.07	7.60	1.14	6.20	1.30
nederig	4.00	1.41	3.38	1.51	5.75	2.05	5.25	3.06	4.88	2.17
nestelen	5.71	1.70	5.14	2.12	5.57	1.27	4.86	2.41	6.57	1.51
netjes	3.29	0.95	5.57	2.37	4.43	2.51	7.43	1.62	7.29	1.25
neurotisch	5.86	1.46	5.14	2.73	7.00	1.15	5.29	2.98	2.57	1.40

nieuws	6.40	1.34	5.40	3.05	6.60	1.82	5.20	1.64	5.80	1.30
nieuwsgierig	7.17	2.14	4.50	3.02	5.83	1.83	5.50	2.74	7.83	1.17
non	2.33	1.97	7.50	1.87	3.17	1.60	8.67	0.52	4.50	2.66
nonchalant	3.56	1.59	4.11	1.90	4.00	1.12	4.00	2.06	5.00	1.66
nutteloos	3.00	2.36	5.30	2.50	4.80	2.10	4.80	2.39	2.30	1.25
nuttig	4.40	1.34	4.40	2.19	5.00	2.45	4.40	1.52	7.60	1.14
obesitas	4.20	3.03	8.00	0.71	5.00	3.39	8.40	1.34	1.60	0.55
obsceen	5.30	2.67	5.50	2.01	6.50	1.90	5.20	2.25	2.50	1.96
obsessie	6.86	2.04	4.43	2.15	7.57	1.40	4.57	3.41	2.71	1.25
oceaan	4.78	2.05	8.22	0.83	6.56	2.24	8.44	0.73	6.56	1.59
octrooi	4.20	2.17	4.80	3.11	3.40	2.30	4.00	3.24	4.80	1.10
oefening	5.13	1.73	5.00	2.39	5.38	1.60	6.00	1.93	6.63	1.30
omhelzen	5.57	2.64	6.71	2.43	5.29	1.89	7.57	2.30	8.43	0.98
omsingeld	5.29	2.06	7.86	1.07	6.29	1.80	7.14	1.95	3.29	0.95
onaangenaam	4.29	2.93	5.14	2.67	6.57	2.82	5.29	2.63	1.71	0.95
onafhankelijkheid	6.86	1.68	6.14	1.35	7.29	1.25	6.29	1.89	7.43	1.27
onbeleefd	3.43	2.07	4.71	2.21	5.00	1.41	4.29	1.80	2.14	0.90
onbeschaamd	6.80	1.30	3.40	1.82	6.20	1.10	3.20	1.30	5.40	1.52
onbezorgd	3.20	2.28	6.20	1.30	3.40	2.30	5.00	1.87	7.20	2.05
onderwijs	4.83	2.40	6.50	2.17	6.50	1.05	6.83	1.72	6.67	0.82
onderworpen	3.67	2.73	4.50	3.51	7.00	3.16	5.83	3.19	1.67	0.82
ongehaast	2.80	1.81	4.70	2.79	3.60	1.71	5.70	2.54	6.80	1.93
ongelukkig	4.40	3.05	4.00	2.35	5.60	2.97	4.80	3.11	1.20	0.45
ongemak	4.00	2.83	5.20	0.84	4.80	1.30	3.40	1.34	2.80	1.10
ongerust	6.00	2.38	4.00	2.08	6.00	1.63	4.86	2.12	3.29	0.95
ongeval	6.11	2.52	6.11	1.45	6.33	1.66	6.11	1.69	2.00	1.12
onhandig	4.60	1.34	6.00	2.12	5.20	2.49	6.60	2.30	4.60	2.19
onheil	6.13	1.13	3.38	2.07	6.25	1.98	5.88	2.17	2.50	1.31
onnozel	4.00	1.00	5.40	1.34	3.40	1.67	4.60	1.67	2.80	0.84
onschuldig	4.29	1.11	4.71	2.06	4.43	1.62	5.43	2.64	6.14	0.90
ontbering	6.57	1.99	5.14	2.54	6.14	1.95	4.14	2.73	2.71	1.60
ontevreden	6.13	2.17	5.00	2.67	6.63	1.60	7.25	1.58	2.50	1.31
ontleden	3.71	1.98	7.29	2.06	6.00	1.15	6.71	1.89	4.14	1.46

ontmoedigd	4.20	2.77	3.00	1.22	5.40	2.70	5.60	1.14	2.20	1.30
ontrouw	6.71	1.70	6.29	2.69	7.43	2.07	6.14	2.67	2.14	1.86
ontspannen	3.83	2.71	3.33	2.58	5.00	2.45	6.50	0.84	8.67	0.52
ontvoering	5.17	3.25	7.00	1.67	7.67	0.82	6.33	3.50	2.50	2.74
ontzaggelijk	4.40	2.22	4.20	2.20	5.90	1.45	3.30	2.06	4.20	1.87
onverschillig	3.60	2.07	5.00	1.87	3.00	1.87	4.40	1.82	3.00	0.71
onvolwassen	4.57	1.72	4.14	1.46	4.14	1.86	5.00	2.38	3.57	0.98
onwetendheid	4.44	2.19	4.78	1.92	4.78	2.11	4.89	2.42	2.44	0.73
onzeker	3.80	2.17	5.60	2.07	3.40	2.61	6.20	2.49	2.60	0.89
onzin	4.88	2.53	3.38	1.69	6.25	1.67	6.25	2.60	3.38	2.26
oorlog	5.14	3.08	7.14	1.57	7.57	1.62	6.71	1.50	1.86	1.46
opgetogen	7.57	1.27	5.86	1.95	6.00	1.91	6.14	2.67	8.43	0.79
opgewekt	7.00	0.71	4.20	1.64	6.20	0.45	5.40	2.07	7.60	0.89
opstand	7.57	1.51	7.29	1.60	7.71	0.95	8.00	1.00	5.29	2.75
optie	4.86	1.86	3.71	2.36	4.86	2.19	4.14	2.61	5.71	0.95
optimisme	6.40	1.52	4.20	1.64	5.60	1.52	5.60	2.07	8.40	0.89
opwinding	9.00	0.00	5.00	3.10	8.33	0.82	5.83	3.43	7.17	1.72
orgasme	7.50	2.81	7.17	2.40	7.33	1.97	8.50	0.84	8.00	1.55
orkaan	6.90	1.52	7.80	1.40	7.10	1.79	8.40	0.70	2.70	1.25
orkest	2.80	2.49	8.80	0.45	4.80	2.59	8.80	0.45	6.20	1.30
overeenstemming	4.71	2.06	5.14	1.57	5.43	1.81	4.86	2.48	6.29	0.76
overlast	5.78	1.79	5.67	2.18	6.56	1.59	5.78	2.22	2.00	0.71
overleden	5.40	2.61	7.20	2.49	3.80	3.03	7.20	2.05	1.80	1.30
overspelig	6.63	1.30	4.50	2.33	6.00	1.41	6.00	3.16	3.13	1.36
overstroming	5.29	2.81	6.57	1.40	5.71	1.50	7.29	2.43	2.00	1.53
overvloed	6.00	1.63	6.57	2.30	6.14	2.04	7.00	2.24	6.43	2.23
overweldigd	7.71	1.38	5.00	1.41	8.00	1.29	5.29	3.09	6.57	1.99
overwinning	7.60	0.55	6.00	2.35	8.40	0.89	6.60	2.70	8.40	0.55
paar	4.00	2.53	6.17	2.79	4.00	2.53	6.83	2.99	6.33	1.21
paard	6.00	2.10	8.67	0.82	7.00	1.79	8.67	0.82	7.67	1.21
paddestoel	2.80	1.62	8.50	0.71	3.50	1.43	8.80	0.42	5.80	1.32
paleis	4.60	2.61	8.40	0.55	5.40	0.89	8.60	0.89	6.80	1.48
pamflet	4.71	2.14	7.14	1.77	5.14	1.68	6.29	1.98	5.43	0.79

paniek	7.44	1.88	5.89	2.20	7.22	1.56	6.89	1.96	1.89	0.93
pannenkoeken	6.00	1.41	8.60	0.89	4.20	2.59	9.00	0.00	7.40	1.52
papier	2.75	2.19	8.88	0.35	3.88	2.23	8.38	1.41	6.25	1.16
paradijs	7.00	2.83	6.71	2.06	6.71	1.38	7.57	1.40	8.71	0.49
paraplu	3.71	1.70	8.43	0.98	4.14	2.19	8.43	1.13	5.29	1.98
parfum	5.75	2.60	7.88	1.46	6.00	1.41	8.25	1.39	7.00	1.20
passie	8.20	0.84	3.80	1.48	6.00	1.41	5.80	1.48	8.60	0.55
pasta	3.33	2.66	7.17	2.64	4.00	2.68	8.83	0.41	7.17	1.33
pastei	2.83	1.94	5.67	3.67	2.83	0.98	6.17	3.06	4.67	1.37
patiënt	3.20	1.48	7.80	1.62	5.10	2.47	7.60	1.84	4.10	2.13
patriot	3.20	2.17	6.00	2.35	4.60	2.30	3.20	1.92	3.80	1.10
penis	7.29	1.25	8.71	0.49	6.71	1.70	8.57	0.53	6.71	1.25
penthouse	5.56	2.13	7.67	1.22	5.78	1.72	7.33	1.50	6.89	1.69
perfectie	5.00	1.22	6.20	1.92	5.20	2.59	4.80	1.64	6.60	1.67
persoon	4.75	1.91	8.13	1.36	6.50	1.93	7.88	1.64	7.13	1.13
pest	4.43	2.94	4.86	2.19	5.29	2.29	5.29	2.43	2.14	1.07
pesten	6.86	2.19	7.14	1.21	8.29	0.76	8.14	1.21	1.14	0.38
pijn	3.43	2.57	7.00	1.53	6.86	2.19	7.43	1.81	1.29	0.76
pistool	7.00	1.00	8.60	0.89	7.80	1.30	9.00	0.00	1.80	1.10
pizza	5.33	1.03	9.00	0.00	5.17	1.33	9.00	0.00	7.67	1.03
plaat	4.00	2.00	5.00	3.74	5.33	1.63	4.83	3.66	5.67	2.07
plagen	4.20	2.78	5.90	2.08	5.50	2.01	6.70	1.77	3.90	1.85
plant	2.40	1.67	8.60	0.89	3.40	1.52	8.20	1.10	6.20	1.64
plechtig	4.00	1.63	3.43	1.62	5.86	1.95	4.14	2.67	4.43	1.40
plezier	6.00	2.06	5.56	2.13	6.89	1.54	6.56	2.24	8.67	0.50
plezierig	7.83	1.83	5.33	2.88	6.83	1.94	5.83	2.56	8.67	0.52
poëzie	3.40	2.61	5.80	2.95	4.60	3.05	6.00	2.55	5.80	2.39
pokken	5.63	1.60	7.63	1.06	6.25	1.04	7.50	1.20	2.00	0.93
pop	4.71	2.21	7.71	1.50	4.86	1.07	7.86	1.46	5.71	1.11
post	4.71	1.70	8.29	0.95	5.29	2.21	8.43	0.79	7.14	1.68
poster	5.14	0.38	8.14	1.57	4.71	1.89	8.14	1.57	5.57	0.98
potlood	1.80	1.79	9.00	0.00	3.40	2.61	9.00	0.00	6.20	1.30
prachtig	6.83	1.60	4.50	1.97	6.50	1.05	5.17	2.48	8.67	0.52

prairie	3.17	2.71	7.17	1.17	3.83	2.56	5.83	3.13	5.33	0.52
prestatie	7.67	1.51	7.00	2.28	7.67	1.97	7.17	2.23	8.17	2.04
prestige	3.60	1.43	3.30	1.64	4.80	2.39	3.00	1.70	5.10	1.85
prettig	6.00	2.31	5.86	2.19	6.43	1.72	6.86	2.48	8.86	0.38
priester	2.80	1.48	8.80	0.45	5.60	1.95	8.40	0.55	4.60	0.89
prikken	5.43	1.72	6.43	2.57	5.71	2.14	6.57	3.05	3.43	1.27
privacy	4.33	1.32	4.78	1.20	6.00	1.32	5.44	1.67	6.89	1.27
problemen	5.40	2.51	5.80	3.11	5.00	2.55	4.40	3.44	2.20	1.64
promotie	7.75	1.04	5.88	2.42	7.13	1.89	6.75	2.05	8.00	0.93
prostituee	4.86	3.34	7.14	1.77	5.86	2.73	7.43	1.13	2.86	1.57
puppy	5.29	3.20	8.14	1.46	5.57	2.94	8.43	0.79	8.14	1.07
pus	3.29	2.63	8.71	0.76	5.86	2.61	9.00	0.00	1.14	0.38
python	7.00	2.35	8.20	1.30	7.80	0.84	8.40	0.89	3.60	1.34
radiator	2.50	1.97	8.83	0.41	3.67	2.16	8.83	0.41	6.50	1.52
radio	6.33	2.07	8.00	0.89	4.83	1.72	8.00	2.00	7.17	1.33
raken	5.40	2.41	5.20	2.66	4.50	1.65	4.70	2.91	5.40	1.58
rammelaar	3.60	2.07	8.80	0.45	4.40	1.52	7.40	3.05	5.80	0.84
ramp	7.57	1.27	5.71	1.89	7.00	1.41	6.14	2.12	1.43	0.53
ranzig	6.13	1.89	5.63	2.88	6.88	1.55	7.25	2.43	2.63	2.72
rat	5.78	1.20	8.44	1.33	5.22	1.92	8.44	1.33	3.22	1.48
rauw	3.80	1.92	7.60	1.67	5.00	2.55	7.60	2.61	3.20	1.79
reünie	5.75	1.83	6.75	1.28	4.50	2.07	7.25	1.16	6.38	1.85
rechtszaak	7.13	1.25	7.50	0.93	7.50	1.07	7.50	1.20	3.25	1.58
redden	6.57	2.30	7.14	1.07	6.43	1.72	6.43	1.90	7.86	1.07
redding	7.43	1.40	7.00	1.53	6.71	2.43	6.43	2.44	8.00	1.15
regen	4.00	1.91	8.43	1.13	5.00	2.08	9.00	0.00	3.86	1.95
regenboog	6.40	1.67	8.40	0.55	4.20	2.28	9.00	0.00	8.20	0.84
reis	7.67	1.21	6.00	2.45	6.17	2.23	6.33	2.25	6.83	1.60
reizen	7.50	1.64	6.67	2.25	5.33	1.86	7.50	1.76	8.00	1.26
rel	6.20	2.10	7.40	1.71	6.20	1.75	7.90	1.29	2.60	1.07
reptiel	4.00	2.74	7.40	1.67	4.60	2.07	8.00	1.73	5.00	1.41
respect	5.57	2.51	4.71	1.89	6.29	2.14	4.86	2.04	8.29	0.76
respectvol	4.89	2.26	4.89	2.03	6.22	1.64	5.33	1.80	8.00	1.00

restaurant	4.20	1.92	9.00	0.00	5.00	2.45	8.60	0.89	6.20	2.39
revolver	5.29	3.30	7.14	1.46	6.57	1.51	8.00	1.15	3.29	1.50
rijkdom	6.29	2.06	6.86	2.54	6.14	1.35	7.14	1.95	7.29	2.06
rijke	5.75	1.67	7.00	1.93	6.75	1.49	7.00	2.20	6.75	1.39
rivier	3.60	1.82	8.40	1.34	3.80	2.17	9.00	0.00	6.20	0.84
roddel	6.33	2.07	4.17	2.32	7.17	1.47	4.83	2.40	2.83	1.33
roestig	2.83	2.14	6.83	1.47	5.17	1.47	7.83	1.17	2.50	1.64
roet	3.00	2.21	7.60	1.65	4.20	2.04	8.10	1.10	3.50	1.08
romantisch	7.29	2.21	5.29	2.93	7.00	1.41	6.29	2.14	8.57	0.79
rommelig	3.40	1.67	4.80	1.79	4.20	1.64	6.40	0.89	5.20	1.30
rood	6.29	2.43	6.71	2.98	8.14	1.07	8.71	0.76	7.00	1.53
rot	4.14	2.27	4.14	2.19	4.43	2.15	4.86	2.48	2.29	0.76
rots	4.44	1.67	7.89	1.90	5.56	2.13	8.22	1.99	5.44	1.24
rotzooi	4.60	2.30	7.60	1.52	5.20	3.11	8.20	1.30	2.00	0.71
rouwdienst	7.50	1.77	6.88	2.30	5.63	2.07	8.00	1.07	1.63	0.92
rover	5.71	2.36	6.71	1.60	6.00	2.00	6.57	1.72	2.86	1.35
ruimte	5.57	1.62	6.43	2.94	5.86	2.04	6.43	3.05	6.71	1.50
ruw	4.00	2.12	7.60	1.52	4.80	2.95	7.60	1.34	4.00	2.35
ruzie	5.75	2.92	6.63	1.60	7.75	1.39	7.63	1.69	1.88	1.13
saffier	5.00	1.73	6.40	2.41	4.00	1.00	5.20	1.92	6.20	0.84
salade	2.00	1.67	8.83	0.41	2.67	2.25	8.83	0.41	6.00	1.10
satijn	6.67	2.07	7.50	1.64	3.83	1.60	6.83	2.64	7.67	1.97
sceptisch	4.20	1.55	5.80	2.70	5.20	2.04	5.20	2.35	4.10	1.79
schaar	1.80	1.30	9.00	0.00	3.80	1.64	9.00	0.00	5.00	0.00
schade	6.43	1.99	6.14	2.04	6.14	1.86	6.29	2.56	2.00	0.82
schaden	5.22	2.64	4.89	2.03	5.44	1.51	5.22	2.11	2.00	0.87
schaduw	4.20	2.28	8.80	0.45	4.00	1.87	8.60	0.89	4.80	1.10
schandaal	7.38	1.30	4.63	2.00	7.63	1.30	6.38	2.26	3.25	2.49
schansspringen	4.86	1.95	6.57	1.27	4.86	1.86	6.71	1.60	5.29	1.38
schat	7.14	1.77	7.00	2.52	7.00	1.63	6.43	2.57	8.57	0.79
schattig	5.71	2.50	6.57	2.07	4.86	2.54	7.29	1.38	7.57	0.98
schedel	2.71	1.80	8.43	1.13	5.14	2.27	8.57	1.13	4.57	0.79
scheermes	3.40	2.07	8.60	0.89	5.60	1.52	9.00	0.00	5.00	1.87

schemering	4.33	1.51	5.83	1.47	4.33	1.97	8.00	0.89	5.67	1.86
scherp	7.33	1.21	6.67	2.42	6.50	2.35	6.00	2.53	6.00	2.37
scherpzinnig	5.57	2.15	5.00	2.00	6.43	1.13	4.43	2.44	7.57	1.51
scheurbuik	3.30	2.71	6.50	2.51	5.30	2.91	4.40	2.76	2.10	1.29
schimmel	4.20	1.30	7.40	2.07	5.60	1.52	7.80	1.79	3.20	1.30
schip	5.00	2.08	8.29	0.76	6.00	1.15	8.71	0.49	6.57	1.62
schoft	6.33	2.55	6.56	1.13	6.33	2.78	7.33	1.12	2.22	1.39
schoonheid	6.60	2.07	6.80	1.92	5.60	2.70	7.20	1.48	8.20	0.84
schoorsteenmantel	4.00	2.33	7.50	2.73	3.38	2.13	7.63	2.72	5.88	0.99
schop	4.00	2.16	7.00	2.00	4.14	1.77	6.86	1.77	4.29	0.95
schorpioen	6.29	1.50	7.71	1.50	5.43	2.37	7.57	1.81	2.71	1.70
schreeuw	6.00	1.53	8.00	1.53	6.71	1.60	8.43	1.51	4.71	2.06
schrijver	5.60	2.30	7.40	1.14	4.40	0.89	7.80	1.30	7.00	1.41
schuim	2.83	2.04	8.33	1.21	3.50	2.17	8.83	0.41	5.50	0.84
schuld	5.00	2.90	6.33	2.66	7.00	2.53	6.17	3.71	1.50	0.84
schuldig	5.29	3.20	6.86	2.04	7.71	1.50	6.29	2.14	2.29	0.76
seks	8.00	1.05	8.30	0.82	7.70	1.42	8.30	0.82	7.80	1.81
sentiment	5.00	2.55	3.40	2.41	4.20	2.39	2.40	1.34	6.00	1.00
sexy	9.00	0.00	5.83	3.54	6.67	2.73	8.00	1.26	8.67	0.82
shotgun	6.86	1.95	7.57	1.27	6.29	2.69	6.43	2.15	2.57	1.72
sjaal	2.78	1.48	7.89	1.62	3.33	2.06	8.33	1.66	6.78	1.56
slaaf	4.80	2.77	7.20	3.03	3.40	2.51	7.40	2.51	1.80	1.30
slachting	7.88	1.36	6.75	2.25	7.88	1.25	7.63	1.19	1.38	0.74
slachtoffer	5.29	3.25	6.29	1.60	6.14	2.41	7.43	1.40	1.29	0.49
slang	6.43	1.99	8.29	1.11	6.86	2.27	7.86	1.86	3.57	1.81
slapen	2.71	1.50	8.71	0.76	5.00	1.29	9.00	0.00	7.14	1.46
slecht	3.90	2.28	4.20	3.22	6.00	2.54	4.10	2.51	1.90	0.88
sleutel	4.20	2.28	8.60	0.89	5.20	2.39	9.00	0.00	5.80	1.92
slib	3.17	2.04	6.33	1.21	5.00	1.26	5.00	2.97	4.67	2.34
slijm	3.67	3.27	6.67	3.01	5.33	3.67	7.33	2.25	2.17	1.33
sloppenwijk	4.40	2.12	7.70	1.64	5.90	1.52	8.40	1.07	2.40	1.35
sluipmoordenaar	7.60	0.55	7.60	2.07	7.20	0.84	7.20	2.95	1.60	0.89
smaak	4.71	2.29	4.14	2.85	5.43	2.57	4.43	3.31	6.29	0.76

smakken	4.67	1.94	6.78	1.48	5.00	1.12	6.89	1.90	2.89	1.05
smalend	3.00	1.87	4.20	2.77	4.00	2.00	3.40	2.30	3.20	1.79
smerig	5.75	2.05	6.38	2.39	6.25	1.75	7.88	1.13	2.38	1.06
sneeuw	6.88	2.53	8.63	0.52	5.88	2.64	8.88	0.35	6.38	2.88
snel	6.00	1.87	6.80	2.39	5.40	1.52	7.00	0.71	7.20	0.84
snelweg	5.14	2.12	8.00	0.82	5.57	2.30	8.57	0.79	5.86	1.86
snijden	6.29	1.11	6.43	2.15	6.57	1.90	7.71	1.98	3.29	1.89
snob	3.63	2.00	6.00	2.67	5.63	2.72	6.00	2.88	3.00	1.31
snoepje	4.00	2.35	9.00	0.00	5.20	2.86	9.00	0.00	6.80	3.35
snoezig	3.50	2.35	4.83	1.60	4.00	1.79	5.67	1.51	6.83	1.83
sociaal	6.17	1.72	4.00	2.90	6.67	0.82	4.67	2.94	7.50	0.84
somber	5.11	2.47	5.44	1.42	5.33	1.58	5.78	1.72	2.11	1.05
somberheid	4.17	2.64	5.50	2.51	5.83	3.13	5.67	3.01	2.00	1.10
speelgoed	3.60	1.78	8.00	0.94	3.90	1.66	8.40	0.70	7.00	1.15
spel	4.40	2.41	7.40	1.14	4.60	1.14	6.20	2.17	7.20	0.84
spin	5.57	2.15	8.57	1.13	5.57	1.99	8.71	0.49	3.71	1.70
spray	4.00	1.94	6.11	1.76	3.78	1.72	6.89	1.54	4.78	0.83
stad	6.60	1.14	8.00	1.73	5.20	2.95	9.00	0.00	6.00	2.45
stagnerend	6.25	1.28	3.50	1.77	5.75	1.39	5.75	2.19	3.00	1.07
stamper	4.00	1.91	6.29	2.21	4.00	1.29	6.43	2.82	4.86	0.90
standbeeld	3.00	1.53	7.14	2.61	4.43	2.30	8.14	1.21	5.57	1.13
stank	2.57	2.44	6.57	2.94	6.86	2.91	8.14	1.21	1.57	0.79
star	4.40	2.41	3.80	3.35	6.00	2.12	4.40	2.97	2.80	1.10
steegje	4.00	2.76	8.33	0.82	5.00	2.61	8.67	0.52	3.83	2.14
ster	7.00	2.10	8.83	0.41	6.00	1.55	9.00	0.00	8.33	1.03
sterfgeval	5.90	2.92	7.50	1.72	7.10	2.23	6.00	3.43	1.50	0.71
sterk	5.80	1.79	7.60	0.89	7.40	1.52	6.80	1.92	8.20	0.84
stijf	4.43	2.07	4.71	1.50	4.71	2.21	5.43	1.90	4.29	1.25
stikken	7.00	2.50	6.33	2.45	7.33	0.87	7.00	1.58	1.56	0.73
stil	3.00	1.26	5.00	2.90	3.67	2.16	5.67	2.73	4.83	0.98
stimulans	6.20	1.92	4.40	1.52	5.00	2.83	4.40	2.61	7.40	1.14
stinken	6.75	1.98	5.75	2.55	7.50	1.41	7.38	1.69	2.38	2.45
stoel	3.43	1.90	8.43	1.51	4.00	1.15	8.14	1.46	6.14	2.04

stoffer	2.43	1.62	7.29	2.06	3.71	2.93	7.00	2.52	4.29	1.25
stom	3.43	1.62	5.29	2.14	5.86	2.73	4.43	2.23	2.71	1.25
storm	6.71	1.50	8.29	1.50	7.29	1.70	8.86	0.38	4.29	1.11
stortplaats	4.40	3.44	8.20	1.30	5.00	3.16	7.80	0.84	2.80	0.84
straat	3.17	2.56	8.67	0.82	4.00	2.45	8.83	0.41	6.00	1.55
straf	4.83	3.19	7.33	1.63	6.50	2.59	7.67	2.34	1.17	0.41
stralend	7.29	1.89	6.14	2.79	7.71	1.70	6.86	2.04	8.43	0.79
strand	5.50	1.58	7.90	1.10	5.10	1.52	8.60	0.97	7.80	1.03
strelen	7.80	0.45	8.40	0.55	4.80	1.79	8.60	0.55	7.60	0.55
streng	3.86	2.19	5.29	2.69	7.00	1.00	5.57	2.07	2.57	1.51
stress	7.71	1.11	4.57	2.23	7.43	1.90	6.00	2.38	2.57	0.79
strop	6.00	1.94	7.11	1.69	5.33	1.73	7.44	1.67	2.11	0.78
succes	6.20	3.03	4.20	2.59	5.00	3.39	3.60	1.52	8.40	0.89
sufferd	4.38	2.00	5.13	1.25	4.88	1.89	5.75	2.19	3.63	1.06
suiker	4.86	2.04	7.57	1.62	5.86	1.46	8.14	1.07	6.71	1.11
suikerriet	2.57	1.62	7.00	2.16	3.57	2.94	6.00	2.83	5.14	0.38
syfilis	4.57	3.46	7.43	1.99	7.14	2.85	6.14	2.34	1.14	0.38
taart	5.40	1.67	8.80	0.45	5.60	2.51	9.00	0.00	8.00	1.73
tabak	4.00	2.10	8.67	0.82	5.00	1.41	7.83	1.33	2.17	1.60
tafel	3.83	2.04	8.83	0.41	6.50	1.64	9.00	0.00	6.00	0.63
talent	6.00	2.00	5.70	2.58	6.00	1.25	5.20	2.25	8.30	0.95
tandarts	5.40	1.14	9.00	0.00	6.00	1.87	9.00	0.00	4.60	1.14
tank	6.00	2.65	8.00	1.53	6.29	2.43	7.71	1.70	3.14	1.68
tartend	5.33	1.94	3.22	1.20	5.11	1.90	3.11	1.54	3.56	1.51
taxi	4.40	1.95	8.80	0.45	4.00	1.73	9.00	0.00	5.20	1.48
teder	4.57	2.70	4.00	1.91	4.57	2.30	6.14	2.27	7.86	1.86
teleurstellen	5.75	1.58	5.50	2.14	6.50	1.51	6.88	1.96	2.75	1.16
tennis	5.57	2.30	7.43	1.27	5.86	1.21	7.71	1.60	5.86	1.57
tepel	5.71	1.38	8.14	1.21	5.71	1.60	8.43	0.98	6.14	1.46
termiet	3.57	1.90	7.86	1.46	6.71	1.70	7.86	1.46	2.86	1.86
terrorist	8.40	0.89	6.60	2.30	8.80	0.45	7.00	1.22	1.00	0.00
tevreden	3.33	2.29	5.67	2.06	5.11	1.69	6.56	1.81	8.56	0.53
theorie	3.17	3.13	5.17	2.86	3.83	3.37	4.00	3.29	6.17	1.60

thermometer	3.00	1.41	8.00	1.55	3.17	1.83	8.17	1.60	5.50	0.84
thuis	5.40	2.63	7.90	1.85	5.90	1.97	8.10	1.60	8.30	1.06
tijd	4.40	1.82	6.20	3.11	6.40	1.82	3.80	3.56	5.20	0.84
tijdschrift	4.43	1.27	8.00	1.15	5.00	2.58	8.29	0.76	7.14	1.57
timide	2.78	1.64	5.00	2.00	1.56	1.33	4.78	2.11	3.89	1.83
toegewijd	3.40	1.82	4.20	1.64	4.20	1.64	3.60	1.14	6.20	2.59
tolerant	3.83	1.72	5.67	2.07	5.33	2.34	5.83	2.64	7.83	1.17
toorn	7.13	1.55	5.13	2.59	6.38	1.06	5.25	2.76	3.25	2.19
toren	3.71	1.60	7.71	0.76	5.14	1.57	8.14	0.90	5.14	0.38
tornado	8.57	0.79	8.71	0.49	8.29	0.76	8.14	1.21	1.71	1.25
tragedie	6.57	2.76	4.00	1.63	6.86	1.35	3.71	2.43	1.29	0.76
trauma	7.40	1.67	6.80	2.17	6.40	2.97	5.60	2.79	1.20	0.45
treurig	6.14	1.68	6.71	1.89	6.14	1.95	7.14	2.12	2.00	1.53
triest	3.33	2.25	5.17	1.94	4.17	2.56	6.83	1.72	1.50	1.22
triomf	7.17	3.13	4.33	2.50	8.17	0.98	5.00	2.37	7.50	1.52
triomfantelijk	6.00	2.45	7.00	3.16	7.67	2.07	6.17	3.37	8.67	0.82
troef	3.40	2.01	4.80	2.44	4.10	1.66	3.90	2.28	5.70	1.16
trofee	3.40	2.07	8.20	0.84	4.00	2.00	8.80	0.45	7.20	0.84
trompet	4.43	1.27	8.14	1.46	6.14	1.46	8.00	1.41	6.00	1.29
trots	6.00	1.00	4.80	1.30	6.20	2.05	5.40	1.95	7.40	1.82
tuin	3.11	1.76	7.89	1.45	4.67	1.80	8.33	1.00	7.33	1.32
tumor	5.40	3.29	8.00	1.22	6.40	2.41	7.20	2.49	1.40	0.89
uil	4.88	1.73	8.75	0.71	4.50	1.93	8.25	1.16	6.88	1.25
uitmuntendheid	6.71	1.38	5.43	2.23	6.71	1.38	3.57	2.15	7.29	1.50
uitschot	5.57	2.70	6.29	1.89	6.00	2.45	4.86	2.41	2.14	1.57
uitstekend	5.29	1.80	6.71	1.89	7.14	1.57	7.00	2.08	8.43	0.79
uitvoering	4.75	2.49	5.25	3.28	5.63	1.41	5.75	2.82	5.75	1.58
urine	1.80	1.79	8.40	1.34	3.20	2.28	9.00	0.00	2.20	0.84
vaandel	3.50	2.95	7.00	3.16	4.00	2.37	6.00	3.22	5.33	0.82
vader	3.67	3.01	8.33	1.21	6.33	2.58	7.50	2.51	7.83	2.04
vadsig	3.20	2.25	5.70	2.67	5.20	2.35	6.80	2.57	2.20	1.03
vagina	5.80	1.30	9.00	0.00	6.40	1.14	8.60	0.89	6.40	1.95
vakantie	6.43	3.10	6.29	2.36	5.57	2.37	7.00	2.24	8.29	0.76

valentijnskaart	6.44	2.24	8.33	0.71	6.22	1.72	8.67	0.50	8.11	1.05
vallen	5.40	2.30	8.20	1.30	3.80	2.39	8.40	0.89	3.60	2.30
vals	6.00	1.58	4.60	1.52	7.80	0.84	5.40	2.70	1.80	0.84
vampier	6.38	2.56	7.25	2.43	5.25	2.43	8.25	1.39	3.25	1.91
vandaal	4.43	2.51	6.00	2.38	5.29	1.60	5.29	2.69	2.14	0.69
varken	4.14	1.86	8.43	0.98	3.14	2.19	8.57	0.79	4.43	1.40
vat	4.14	2.27	8.86	0.38	5.71	1.25	8.71	0.76	5.29	0.76
vechten	8.20	0.84	7.00	1.22	8.00	0.71	7.00	2.55	1.80	0.84
veilig	2.33	1.21	4.00	2.10	4.50	1.97	5.17	2.64	8.50	0.84
veld	4.83	1.60	7.33	1.97	6.00	3.16	7.83	1.17	6.83	0.75
venster	2.40	1.71	8.00	1.70	3.50	2.22	8.20	1.75	5.10	0.88
verachten	6.00	1.73	5.80	1.64	6.60	2.07	2.60	0.55	2.00	0.71
verafschuwen	6.86	2.12	4.14	2.61	6.57	2.44	4.43	2.94	1.43	0.79
verandering	7.11	1.36	5.89	1.27	6.11	1.17	5.33	1.58	6.44	1.13
verbaasd	4.80	1.30	5.00	2.00	4.40	1.52	6.40	1.52	5.00	1.22
verbergen	6.25	1.39	4.25	2.66	6.00	1.60	6.00	3.12	4.00	1.31
verbeteren	4.29	1.60	5.29	2.06	5.00	1.15	4.43	2.37	5.29	0.95
verblinding	6.00	1.73	6.86	1.95	4.86	2.27	6.43	2.30	2.43	1.13
verdacht	6.00	2.97	4.50	2.35	6.17	0.75	4.00	2.83	2.00	0.89
verdorven	5.71	1.80	3.00	1.41	5.57	2.23	3.29	1.60	2.86	1.95
verdovend	3.63	1.92	6.00	2.62	6.38	1.60	5.63	2.39	3.75	1.04
verdriet	5.80	1.92	5.80	1.10	4.60	2.51	7.80	0.84	2.00	1.41
verdrinken	6.17	2.99	6.50	2.51	5.50	2.17	7.67	1.21	1.17	0.41
verf	4.67	2.34	8.50	0.55	5.33	2.42	9.00	0.00	7.00	1.79
verfrissing	4.70	1.89	6.10	2.56	4.30	1.34	6.20	2.66	7.40	1.17
vergif	5.40	2.61	6.60	3.21	5.80	2.95	6.20	3.11	1.20	0.45
vergissing	5.29	1.70	4.14	1.68	5.00	1.53	4.71	2.43	3.71	1.11
verhongeren	5.67	2.87	6.22	2.22	5.44	2.40	6.78	2.28	1.78	0.83
verjaardag	7.60	1.67	8.40	0.89	5.20	2.86	8.20	1.30	7.80	1.64
verkrachting	7.38	2.83	7.25	2.38	7.50	2.73	6.88	1.64	1.00	0.00
verkwistend	3.43	2.37	4.00	2.31	3.57	1.90	4.29	2.14	2.86	1.46
verlamming	6.71	2.14	7.00	1.83	6.71	1.60	7.43	1.90	1.29	0.76
verlangen	6.71	0.49	5.43	2.37	8.00	1.15	4.00	2.71	5.86	1.21

verlegen	2.40	1.14	3.80	1.64	2.40	1.95	6.00	1.58	4.20	1.30
verlegenheid	3.17	1.33	5.00	2.00	3.67	2.42	7.17	1.72	4.67	1.63
verloren	4.00	2.97	4.50	3.45	4.83	3.37	5.50	3.56	1.67	0.82
verlosser	3.90	2.42	5.10	2.69	4.80	2.39	5.60	2.50	5.90	1.52
verminken	5.40	2.70	6.80	1.10	4.60	3.05	6.80	1.79	1.20	0.45
vermoeid	3.29	1.50	4.14	2.41	4.71	2.14	4.86	3.13	2.43	0.98
vernederen	5.89	2.47	5.89	1.54	6.89	2.03	6.33	1.73	1.67	0.71
vernietigen	6.40	2.51	6.80	1.48	4.80	3.49	6.80	2.28	1.80	1.10
vernietiging	7.38	1.92	5.88	2.36	7.50	2.00	7.00	2.33	1.75	1.04
verontreinigen	3.57	2.30	5.43	2.30	4.71	2.06	6.29	1.98	2.43	1.27
verpleegkundige	4.14	1.68	8.14	1.46	5.14	2.54	8.29	1.25	6.00	1.41
verpletterd	7.43	0.98	5.86	1.46	7.86	1.21	5.00	1.15	4.00	2.38
verraden	6.40	2.07	4.40	2.07	7.80	1.10	4.60	2.61	1.60	0.89
verrader	6.67	2.58	6.17	1.72	6.17	1.83	5.50	3.56	1.33	0.52
verrast	8.00	1.10	4.83	2.79	6.50	1.87	6.33	3.44	7.50	1.22
verrot	5.22	2.49	5.44	1.94	4.67	2.65	5.67	2.12	1.89	0.93
verrukking	5.70	2.31	5.20	2.49	5.60	1.07	5.10	3.07	7.60	1.26
verschrikkelijk	5.60	1.82	3.20	2.28	6.80	1.30	4.00	2.00	1.80	0.84
verschrikking	6.86	1.95	4.57	2.64	6.57	2.30	4.43	2.57	2.29	1.11
verschroeiend	5.89	1.76	4.78	2.11	5.78	1.64	4.00	2.29	2.89	0.78
versieren	6.00	2.24	7.00	1.87	5.20	2.49	6.80	1.79	7.20	1.79
verslaafd	5.63	2.62	4.88	2.30	6.50	1.85	6.63	2.67	3.00	2.07
verslaafde	4.43	3.36	6.43	2.37	6.00	1.41	6.14	2.34	2.14	1.21
verslagen	4.80	2.39	4.60	1.95	4.80	2.59	6.80	1.92	2.60	1.52
verstand	3.86	2.19	6.29	2.81	6.00	1.91	7.14	1.77	7.71	1.80
verstoren	5.14	1.77	6.57	1.90	5.86	2.19	5.29	2.87	3.14	1.35
vertraagd	3.00	2.45	4.00	2.12	4.80	3.03	5.60	2.07	2.60	0.89
vertrouwen	5.17	1.72	4.17	2.48	5.83	1.33	4.67	2.88	8.67	0.52
verveeld	3.80	2.39	4.20	2.05	5.20	1.64	5.80	1.92	3.00	1.41
verveling	2.33	1.63	3.83	2.79	4.67	2.50	5.33	3.20	3.33	2.42
verwaarloosd	4.40	2.12	5.00	2.31	5.30	2.41	5.60	2.55	1.70	0.82
verwaarlozing	5.40	2.30	5.00	2.00	5.20	2.59	7.00	1.87	2.00	1.00
verward	5.57	2.23	3.86	2.04	4.57	2.82	4.29	2.29	3.57	1.51

verwijfd	5.00	2.18	5.33	1.22	6.33	1.66	6.22	1.86	3.67	1.41
verwonding	5.40	1.82	7.00	1.22	3.80	1.79	6.80	1.92	1.80	0.84
verworpen	5.38	2.13	4.25	1.49	5.38	1.41	3.75	1.49	3.13	1.46
vest	3.14	1.68	7.86	1.46	4.29	1.70	8.57	0.79	7.00	1.53
vet	3.00	1.58	6.40	2.19	7.20	1.48	7.40	2.51	2.60	1.52
vierkant	3.14	1.68	7.29	2.36	4.29	2.56	7.57	2.30	5.86	2.27
vies	5.44	2.07	7.00	1.22	5.67	1.87	8.11	0.60	2.33	0.87
viezerik	3.43	2.76	7.14	1.57	6.71	2.69	7.43	1.40	1.86	0.90
vijandig	6.00	1.58	6.40	1.52	5.20	2.59	5.00	2.35	2.60	1.14
vinger	3.20	1.64	8.40	1.34	3.40	2.07	9.00	0.00	6.20	1.10
viool	3.33	2.66	9.00	0.00	4.17	2.56	8.83	0.41	6.50	1.76
vis	3.33	2.58	8.17	0.98	4.33	3.08	9.00	0.00	6.67	1.51
visie	4.30	2.50	4.30	2.71	5.50	2.27	3.50	2.46	6.40	1.43
vlag	1.80	1.10	9.00	0.00	3.20	2.17	9.00	0.00	5.00	0.00
vlak	2.60	1.52	6.00	4.12	2.60	1.14	6.20	3.83	4.60	0.89
vlakte	4.00	1.29	5.71	2.14	4.14	1.35	6.43	2.70	4.43	0.79
vlinder	3.33	2.35	8.78	0.44	4.33	1.50	8.67	0.50	7.33	0.71
vlug	5.71	0.95	5.57	1.99	5.71	0.95	6.29	2.43	6.29	0.95
voeden	3.60	1.34	6.40	2.97	3.80	2.28	7.40	1.82	6.40	1.52
voedsel	5.88	2.23	7.63	1.51	6.88	1.25	7.88	1.73	8.25	0.71
voertuig	4.00	2.52	7.71	1.11	6.71	1.70	8.00	1.15	6.43	1.62
voet	2.86	1.21	8.29	1.50	2.71	1.98	8.57	0.79	5.57	0.98
vogel	4.29	2.36	8.86	0.38	4.14	2.61	8.86	0.38	6.14	1.21
volwassen	3.40	2.51	6.80	2.28	6.60	1.67	5.40	2.30	6.60	1.52
voordeel	5.33	1.21	4.17	2.48	5.17	1.33	4.33	1.97	7.83	0.98
voorn	3.33	1.86	4.17	3.25	4.67	2.25	3.50	3.27	4.67	0.82
vooruitgang	5.00	1.83	5.50	2.92	5.30	1.64	5.20	1.87	8.00	0.94
vork	2.80	1.64	9.00	0.00	5.00	1.41	9.00	0.00	5.60	1.34
vormen	5.14	1.57	3.29	2.14	5.43	1.81	4.43	2.15	6.00	1.15
vrachtwagen	3.78	2.05	8.89	0.33	5.89	2.20	8.78	0.67	5.33	1.73
vrede	4.40	3.29	4.00	2.83	6.00	2.74	5.60	3.85	8.20	1.10
vreemd	5.43	1.40	4.71	1.89	5.71	1.38	5.29	2.29		0.90
vreemdeling	5.75	1.49	6.00	1.60	5.25	1.83	7.00	1.85	3.75	2.19

vreselijk	3.57	2.07	3.57	1.13	5.43	2.15	4.43	1.99	2.00	1.00
vreugde	6.29	2.69	6.00	2.24	6.43	1.51	5.43	2.64	8.14	1.21
vreugdevol	4.80	1.79	5.20	3.11	5.20	2.39	6.80	1.30	7.60	1.14
vriend	6.43	1.81	7.86	1.07	6.71	1.80	8.00	1.00	8.86	0.38
vriendelijk	4.80	2.49	4.20	2.05	6.00	1.00	5.80	2.77	8.20	0.84
vriendelijkheid	5.43	2.70	6.29	2.14	4.71	2.36	8.00	1.00	8.43	0.98
vriesvak	2.60	1.82	8.60	0.89	3.40	2.30	9.00	0.00	4.20	1.10
vrij	6.17	2.04	5.33	2.73	7.33	1.37	6.17	2.79	8.83	0.41
vrijheid	7.50	1.52	6.67	2.25	7.00	1.67	6.83	2.71	9.00	0.00
vrijstaand	3.10	1.66	6.40	1.90	4.20	2.04	6.00	1.89	6.30	1.34
vrouw	4.80	2.86	8.80	0.45	5.60	0.89	9.00	0.00	7.80	1.79
vuil	5.25	2.43	6.75	1.91	6.25	1.16	8.13	1.13	3.25	1.98
vuiligheid	4.86	1.86	5.29	2.06	5.57	1.62	5.71	2.56	3.14	1.21
vuilnis	1.33	0.82	6.17	4.02	4.50	2.66	7.50	3.21	2.17	1.33
vulkaan	6.44	1.88	8.44	0.88	6.22	1.64	8.44	0.88	4.89	1.17
vuurtoren	3.80	2.59	9.00	0.00	4.20	2.28	9.00	0.00	5.80	1.79
vuurwapen	8.00	1.77	8.50	0.93	7.50	1.85	8.50	0.93	1.50	1.07
vuurwerk	7.00	1.53	7.86	1.21	7.29	1.38	8.43	0.79	7.43	1.51
waardig	4.38	1.19	4.88	2.30	6.13	0.83	6.13	2.75	7.25	1.16
waarheid	7.29	0.49	4.86	3.08	7.71	1.50	5.14	3.13	7.14	2.04
wagen	5.00	3.00	6.86	2.85	5.57	2.51	6.43	3.36	6.00	1.00
walgen	7.20	1.30	4.00	2.00	6.80	2.28	5.80	1.30	1.00	0.00
wanhopig	4.90	2.60	4.90	2.51	5.30	2.87	5.90	2.13	1.70	0.82
wapen	6.33	2.16	8.83	0.41	7.00	3.10	8.83	0.41	2.50	1.52
warmte	7.50	1.76	8.00	1.10	7.67	1.03	7.00	2.90	8.83	0.41
water	3.20	2.20	8.70	0.67	5.00	2.36	8.70	0.48	6.80	1.14
waterkoker	1.80	1.10	9.00	0.00	3.20	1.79	9.00	0.00	5.40	0.89
waterval	5.14	2.54	7.29	1.98	4.43	1.13	8.43	0.53	7.29	1.38
weelderig	5.00	1.87	4.56	2.07	5.56	1.59	5.33	2.50	6.67	1.80
wensen	6.00	1.41	6.60	1.67	4.00	1.73	5.20	1.92	6.20	1.64
wereld	6.63	1.19	8.00	1.51	7.13	2.64	8.25	1.16	7.00	2.33
werkgelegenheid	4.43	1.90	6.57	1.62	6.29	1.25	5.14	2.19	6.29	1.11
werktuig	4.14	2.34	7.14	1.57	3.57	2.30	6.57	2.70	4.86	1.46

wesp	4.86	2.67	9.00	0.00	6.86	1.77	9.00	0.00	3.43	1.72
wijn	6.00	1.00	9.00	0.00	5.00	0.71	9.00	0.00	7.60	1.14
wijs	3.60	1.67	6.00	1.00	5.20	2.17	4.40	2.30	8.20	1.10
wind	4.00	2.83	7.83	1.83	4.00	2.37	7.83	1.17	5.17	1.83
windmolen	4.17	1.17	8.67	0.82	6.00	2.19	9.00	0.00	6.83	1.33
winnen	6.70	0.95	7.20	1.87	6.80	1.48	7.30	2.31	7.90	0.88
winst	5.80	1.79	8.20	0.45	6.40	1.14	7.20	1.64	8.00	1.00
wit	3.71	1.70	7.57	1.81	5.57	1.27	8.00	1.73	6.14	1.07
woede	8.11	0.78	5.67	1.87	7.33	1.22	6.67	1.50	2.44	1.33
woedend	6.14	2.61	5.00	2.58	7.29	1.80	7.57	1.13	1.57	0.79
wolken	4.40	2.61	9.00	0.00	5.00	2.45	9.00	0.00	7.00	2.00
wolkenkrabber	5.63	2.00	8.75	0.71	6.00	2.07	8.75	0.46	5.88	1.25
wonden	4.86	2.79	6.43	1.62	5.29	1.70	7.57	1.13	2.00	1.00
wonder	7.57	1.51	6.57	2.23	7.00	1.00	5.57	2.76	8.00	0.82
wreed	7.00	1.83	4.14	2.79	6.57	2.64	4.86	3.24	1.71	0.95
wringer	4.00	1.53	6.29	2.21	4.29	2.75	5.86	2.79	4.86	0.38
zacht	4.29	2.63	4.86	2.85	4.43	2.15	5.71	2.50	7.43	0.79
zachtmoedig	4.00	1.00	4.86	1.95	4.57	0.98	4.00	1.73	6.43	1.81
zeeziek	4.80	2.68	7.00	1.00	4.40	2.07	8.00	1.00	2.00	1.22
zegenen	3.17	2.14	5.00	2.53	4.17	2.48	5.67	2.66	6.50	1.87
zeilboot	6.67	2.07	8.33	0.82	6.50	1.97	9.00	0.00	8.00	1.55
zeker	4.60	1.78	4.80	2.97	5.90	2.51	3.70	2.63	7.80	0.79
zelfmoord	6.60	3.21	7.80	1.79	6.80	3.35	7.80	2.68	1.00	0.00
zelfverzekerd	6.00	2.77	4.43	2.57	7.43	1.81	5.71	2.43	7.71	0.76
zenuwachtig	7.22	1.39	5.67	2.12	6.56	1.59	6.44	2.01	3.22	1.30
ziek	4.50	3.62	6.83	2.32	5.00	3.41	8.17	0.98	1.33	0.52
ziekenhuis	6.80	1.30	9.00	0.00	6.40	3.13	8.40	0.89	6.00	2.83
ziekte	5.25	1.16	5.63	2.67	7.00	1.60	7.75	1.16	1.50	0.76
ziektekiemen	4.14	2.61	5.14	2.19	4.57	1.40	4.00	1.41	2.57	2.07
zitbank	2.00	1.15	8.71	0.76	4.43	2.94	8.57	0.79	6.86	1.46
zitkruk	2.88	1.89	8.13	1.46	3.38	1.69	8.13	1.46	5.13	0.35
zitplaats	3.40	2.07	7.80	1.79	4.80	3.03	9.00	0.00	7.00	1.58
zomerhuisje	5.17	2.71	8.17	0.98	4.50	2.43	8.17	1.33	7.83	0.98

zon	8.50	0.84	9.00	0.00	7.83	1.94	8.50	1.22	9.00	0.00
zonde	4.20	2.10	4.60	2.46	5.50	1.96	3.90	2.33	2.60	0.84
zondebok	4.20	2.39	5.80	1.79	4.60	2.30	5.40	2.30	2.00	1.00
zondig	5.71	1.60	4.71	2.43	5.29	1.89	4.86	2.61	2.86	1.21
zonlicht	4.33	2.29	8.11	0.60	5.67	1.66	8.56	0.73	8.56	0.73
zonsondergang	6.20	2.77	8.60	0.89	4.40	2.88	8.00	1.73	7.40	1.34
zonsopkomst	5.25	2.55	8.38	0.92	5.13	2.47	8.75	0.46	8.25	0.71
zorgeloos	3.43	2.37	5.43	2.37	5.86	2.04	5.00	1.73	7.57	1.51
zuigeling	3.86	2.85	6.71	2.14	4.86	2.73	8.00	1.53	6.29	1.80
zuur	4.14	2.34	7.71	0.95	5.29	2.21	8.29	0.76	3.43	1.13
zwachtel	4.20	2.59	8.20	0.84	3.20	1.79	8.20	0.84	3.00	1.00
zwak	2.50	2.51	5.00	2.53	6.00	2.83	6.17	2.48	1.67	1.21
zwart	3.17	2.56	7.83	2.04	4.83	2.56	9.00	0.00	4.67	1.03
zwemmer	7.00	2.28	7.00	2.10	5.17	1.72	8.50	0.84	8.83	0.41

Appendix C: Main results from Experiments 1-3 presented as weighted scatter plots of Right Side Advantage predicting Valence.

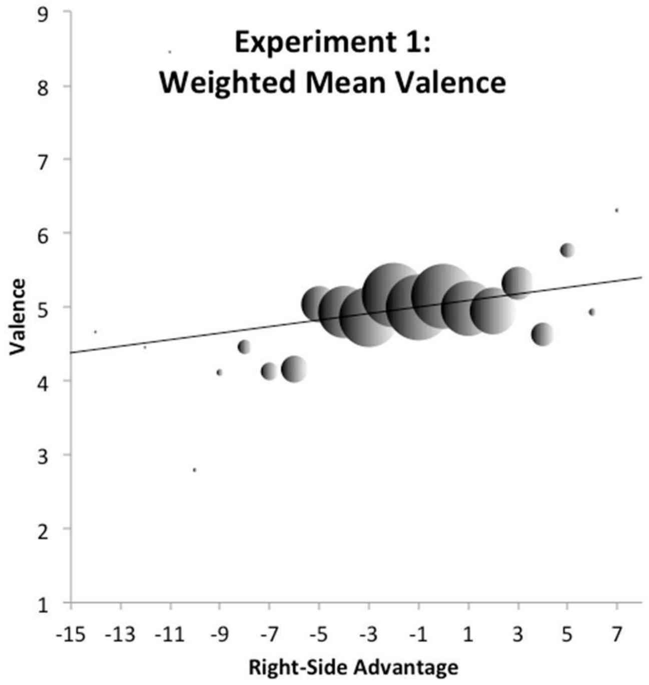

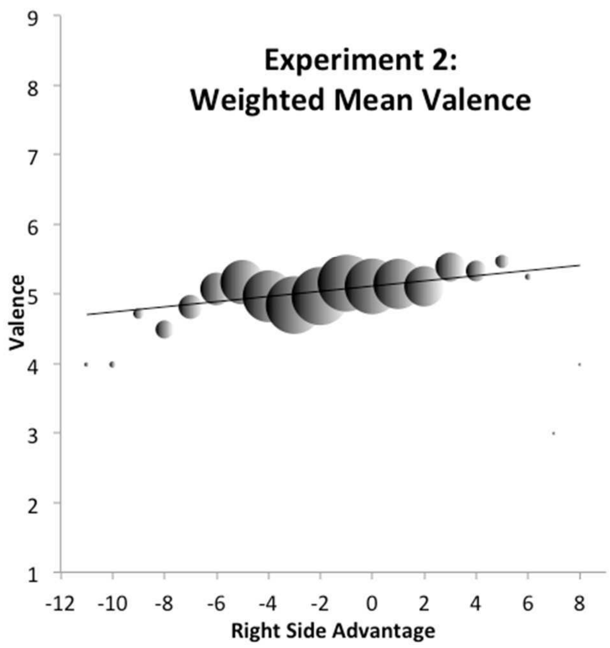

Experiment 2:
Weighted Mean Valence
Valence
Right Side Advantage
9
8
7
6
5
4
3
2
1
-12
-10
-8
-6
-4
-2
0
2
4
6
8

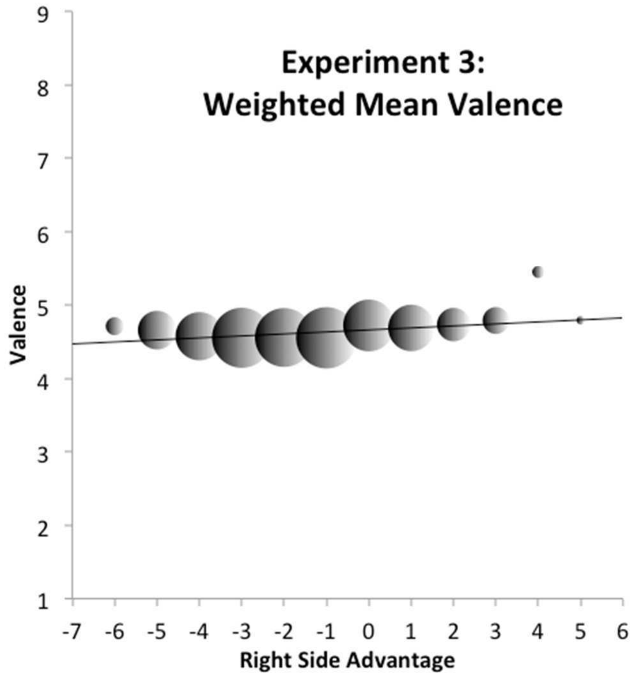
Experiment 3:
Weighted Mean Valence
Valence
9
8
7
6
5
4
3
2
1
-7 -6 -5 -4 -3 -2 -1 0 1 2 3 4 5 6
Right Side Advantage

Appendix D: List of words from the AFINN corpus which post-dated the invention of the QWERTY keyboard.

apesh*t
a**f**king
a**hole
bada**
blockbuster
brainwashing
bullsh*t
c**cksucker
c**cksuckers
dickhead
dipsh*t
douchebag
dumba**
fraudster
fraudsters
frikin
ftw
f**kface
f**khead
f**ktard
f*ked
f*king
green wash
green washing
greenwash
greenwasher
greenwashers
greenwashing
lawl
lmao
lmfao
lol
motherf**ker
motherf**king
n00b
n*ggas
noob
oks
prblm
prblms
rofl
roflcopter
roflmao

rotfl

rotflmfao
scumbag
sh*thead
sh*tty
smog
spam
spammer
spammers
spamming
tard
wanker
woohoo
wooo
woow
wowow
wowww
wtf
yeees
yucky

Appendex E: Complete list of words in the Pseudoword corpus.

bague
bashe
bave
beeg
beesh
beeve
blabe
blafe
blage
blague
blale
blashe
blathe
blave
bleeb
bleef
bleeg
bleege
bleel
bleesh
bleeth
bleeve
blobe
blofe
bloge
blogue
blole
bloob
bloof
bloog
blooge
blool
bloosh
blooth
bloove
bloshe
blothe
blove
bogue
boog
boosh
boove
boshe
bove
brabe
brage
brague
brape
brare
brashe
brathe
breeb
breeg
breege
breep
breer
breesh
breeth
brobe
broge
brogue
broob
broog
brooge
broop
broor
broosh
brooth
brope
brore
broshe
brothe
chabe
chade
chage
chague
chale
chame
chathe
cheeb
cheed
cheeg
cheege
cheel
cheem
cheeth
chobe
chode
choge
chogue
chole
chome
choob
chood
choog
chooge
chool
choom
chooth
chothe
dabe
dache
dafe
dague
dathe
deeb
deech
deef
deeg
deeth
dobe
doche
dofe
dogue
doob
dooch
doof
doog
dooth
dothe
drabe
drache
drade
drafe
drage
drague
drare
drase
drate
drathe
draze
dreeb
dreech
dreed
dreef
dreeg
dreege
dreer
dreese
dreet
dreeth
dreeze
drobe
droche
drode
drofe
droge
drogue
droob
drooch
drood
droof
droog
drooge
droor
droose
droot
drooth
drooze
drore
drose
drote
drothe
droze
dwache
dwade
dwafe
dwage
dwague
dwake
dwale
dwame
dwane
dwape
dware
dwase
dwashe
dwate
dwathe
dwave
dwaze
dweech
dweed
dweef
dweeg
dweege
dweek
dweel
dweem
dween
dweep
dweer
dweese
dweesh
dweet
dweeth
dweeve
dweeze
dwoche
dwode
dwofe
dwoge
dwogue
dwoke
dwole
dwome
dwone
dwooch
dwood
dwoof
dwoog
dwooge
dwook
dwool
dwoom
dwoon
dwoop
dwoor
dwoose
dwoosh
dwoot
dwooth
dwoove
dwooze
dwope
dwore
dwose
dwoshe
dwote
dwothe
dwove
dwoze
fabe
fache
fafe
fague
fape
feeb
feech
feef
feeg
feep
flabe
flache
flafe
flage
flague
flape
flashe
flathe
flave
fleeb
fleech
fleef
fleeg
fleege
fleep

fleesh
fleeth
fleeve
flobe
floche
flofe
floge
flogue
floob
flooch
floof
floog
flooge
floop
floosh
flooth
floove
flope
floshe
flothe
flove
fobe
foche
fofe
fogue
foob
fooch
foof
foog
foop
fope
frabe
frache
frafe
frage
frague
frape
frashe
frathe
frave
freeb
freech
freef
freeg
freege
freep

freesh
freeth
freeve
frobe
froche
frofe
froge
frogue
froob
frooch
froof
froog
frooge
froop
froosh
frooth
froove
frope
froshe
frothe
frove
gache
gathe
geech
geeth
glache
glafe
glage
glague
glake
glale
glase
glashe
glathe
gleech
gleef
gleeg
gleege
gleek
gleel
gleese
gleesh
gleeth
gloche
glofe
gloge

glogue
gloke
glole
glooch
gloof
gloog
glooge
glook
glool
gloose
gloosh
glooth
glose
gloshe
glothe
goche
gooch
gooth
gothe
grabe
grache
grage
grague
grare
grashe
greeb
greech
greeg
greege
greer
greesh
grobe
groche
groge
grogue
groob
grooch
groog
grooge
groor
groosh
grore
groshe
gwabe
gwache
gwade

gwafe
gwage
gwague
gwake
gwale
gwame
gwane
gwape
gware
gwase
gwashe
gwate
gwathe
gwave
gwaze
gweeb
gweech
gweed
gweef
gweeg
gweege
gweek
gweel
gweem
gween
gweep
gweer
gweese
gweesh
gweet
gweeth
gweeve
gweeze
gwobe
gwoche
gwode
gwofe
gwoge
gwogue
gwoke
gwole
gwome
gwone
gwoob
gwooch
gwood

gwoof
gwoog
gwooge
gwook
gwool
gwoom
gwoon
gwoop
gwoor
gwoose
gwoosh
gwoot
gwooth
gwoove
gwooze
gwope
gwore
gwose
gwoshe
gwote
gwothe
gwove
gwoze
jabe
jache
jafe
jague
jame
jashe
jate
jathe
jeeb
jeech
jeef
jeeg
jeem
jeesh
jeet
jeeth
jobe
joche
jofe
jogue
jome
joob
jooch

joof
joog
joom
joosh
joot
jooth
joshe
jote
jothe
kabe
kague
keeb
keeg
klabe
klache
klade
klafe
klague
klale
klape
klashe
kleeb
kleech
kleed
kleef
kleeg
kleel
kleep
kleesh
klobe
kloche
klode
klofe
klogue
klole
kloob
klooch
klood
kloof
kloog
klool
kloop
kloosh
klope
kloshe
kobe

kogue
koob
koog
krabe
krache
krafe
krage
krague
krare
krashe
krathe
kreeb
kreech
kreef
kreeg
kreege
kreer
kreesh
kreeth
krobe
kroche
krofe
kroge
krogue
kroob
krooch
kroof
kroog
krooge
kroor
kroosh
krooth
krore
kroshe
krothe
kwabe
kwache
kwade
kwage
kwague
kwame
kwape
kwase
kwashe
kwave
kwaze
kweeb
kweech
kweed
kweeg
kweege
kweem
kweep
kweese
kweesh
kweeve
kweeze
kwobe
kwoche
kwode
kwoge
kwogue
kwome
kwoob
kwooch
kwood
kwoog
kwooge
kwoom
kwoop
kwoose
kwoosh
kwoove
kwooze
kwope
kwose
kwoshe
kwove
kwoze
lache
lale
lashe
leech
leel
leesh
loche
lole
looch
lool
loosh
loshe
mabe
mafe
mashe
mathe
meeb
meef
meesh
meeth
mobe
mofe
moob
moof
moosh
mooth
moshe
mothe
nabe
nache
nafe
nage
nague
nashe
nathe
neeb
neech
neef
neeg
neege
neesh
neeth
nobe
noche
nofe
noge
nogue
noob
nooch
noof
noog
nooge
noosh
nooth
noshe
nothe
pabe
pague
pame
pashe
pathe
peeb
peeg
peem
peesh
peeth
plache
plafe
plage
plake
plale
plape
plare
plashe
plathe
plave
pleech
pleef
pleege
pleek
pleel
pleep
pleer
pleesh
pleeth
pleeve
ploche
plofe
ploge
ploke
plole
plooch
ploof
plooge
plook
plool
ploop
ploor
ploosh
plooth
ploove
plope
plore
ploshe
plothe
plove
pobe
pogue
pome
poob
poog
poom
poosh
pooth
poshe
pothe
prache
prage
prake
prale
prame
prape
prashe
prate
prathe
preech
preege
preek
preel
preem
preep
preesh
preet
preeth
proche
proge
proke
prole
prome
prooch
prooge
prook
prool
proom
proop
proosh
proot
prooth
prope
proshe
prote
prothe
rashe
reesh
roosh
roshe
sabe
sache
sague
sashe
seeb
seech
seeg
seesh
shabe
shache
shage
shague
shase
sheeb
sheech
sheeg
sheege
sheese
shobe
shoche
shoge
shogue
shoob
shooch
shoog
shooge
shoose
shose
shrabe
shrache
shrafe
shrage
shrague
shrale
shrame
shrane
shrape
shrare
shrase
shrashe
shrate

shrathe
shrave
shraze
shreeb
shreech
shreef
shreeg
shreege
shreel
shreem
shreen
shreep
shreer
shreese
shreesh
shreet
shreeth
shreeve
shreeze
shrobe
shroche
shrofe
shroge
shrogue
shrole
shrome
shrone
shroob
shrooch
shroof
shroog
shrooge
shrool
shroom
shroon
shroop
shroor
shroose
shroosh
shroot
shrooth
shroove
shrooze
shrope
shrore
shrose
shroshe
shrote
shrothe
shrove
shroze
skabe
skafe
skage
skague
skake
skame
skase
skashe
skathe
skave
skeeb
skeef
skeeg
skeege
skeek
skeem
skeese
skeesh
skeeth
skeeve
skobe
skofe
skoge
skogue
skoke
skome
skoob
skoof
skoog
skooge
skook
skoom
skoose
skoosh
skooth
skoove
skose
skoshe
skothe
skove
slabe
slache
slade
slafe
slage
slague
slale
slame
slare
slase
slashe
sleeb
sleech
sleed
sleef
sleeg
sleege
sleel
sleem
sleer
sleese
sleesh
slobe
sloche
slode
slofe
sloge
slogue
slole
slome
sloob
slooch
slood
sloof
sloog
slooge
slool
sloom
sloor
sloose
sloosh
slore
slose
sloshe
smabe
smade
smafe
smage
smague
smale
smame
smane
smape
smase
smashe
smathe
smave
smaze
smeeb
smeed
smeef
smeeg
smeege
smeel
smeem
smeen
smeep
smeese
smeesh
smeeth
smeeve
smeeze
smobe
smode
smofe
smoge
smogue
smole
smome
smone
smoob
smood
smoof
smoog
smooge
smool
smoom
smoon
smoop
smoose
smoosh
smooth
smoove
smooze
smope
smose
smoshe
smothe
smove
smoze
snabe
snache
snade
snafe
snage
snague
sname
snane
snase
snashe
snathe
snave
sneeb
sneech
sneed
sneef
sneeg
sneege
sneem
sneen
sneese
sneesh
sneeth
sneeve
snobe
snoche
snode
snofe
snoge
snogue
snome
snone
snoob
snooch
snood
snoof
snoog
snooge
snoom
snoon
snoose
snoosh
snooth
snoove
snose
snoshe
snothe
snove
sobe
soche
sogue
soob
sooch
soog
soosh
soshe
spabe
spage
spague
spame
spape
spashe
spate
spathe
spave
speeb
speeg
speege
speem
speep
speesh
speet
speeth
speeve
spobe
spoge
spogue
spome
spoob
spoog
spooge
spoom
spoop
spoosh
spoot

spooth
spoove
spope
sposhe
spote
spothe
spove
stabe
stache
stafe
stague
stase
stashe
stathe
steeb
steech
steef
steeg
steese
steesh
steeth
stobe
stoche
stofe
stogue
stoob
stooch
stoof
stoog
stoose
stoosh
stooth
stose
stoshe
stothe
swabe
swache
swafe
swage
swague
swale
swame
swase
swathe
swave
swaze

sweeb
sweech
sweef
sweeg
sweege
sweel
sweem
sweese
sweeth
sweeve
sweeze
swobe
swoche
swofe
swoge
swogue
swole
swome
swoob
swooch
swoof
swoog
swooge
swool
swoom
swoose
swooth
swoove
swooze
swose
swothe
swove
swoze
tafe
tage
tague
tashe
tave
teef
teeg
teege
teesh
teeve
thabe
thache
thade

thafe
thage
thague
thake
thale
thane
thape
thashe
thate
thathe
thave
theeb
theech
theed
theef
theeg
theege
theek
theel
theen
theep
theesh
theet
theeth
theeve
thobe
thoche
thode
thofe
thoge
thogue
thoke
thole
thone
thoob
thooch
thood
thoof
thoog
thooge
thook
thool
thoon
thoop
thoosh
thoot

thooth
thoove
thope
thoshe
thote
thothe
thove
thrabe
thrache
thrade
thrafe
thrage
thrague
thrake
thrale
thrame
thrape
thrare
thrashe
thrathe
thrave
threeb
threech
threed
threef
threeg
threege
threek
threel
threem
threep
threer
threesh
threeth
threeve
throbe
throche
throde
throfe
throge
throgue
throke
throle
throme
throob
throoch

throod
throof
throog
throoge
throok
throol
throom
throop
throor
throosh
throoth
throove
thrope
throre
throshe
throthe
throve
thwabe
thwache
thwade
thwafe
thwage
thwague
thwake
thwale
thwame
thwane
thwape
thware
thwase
thwashe
thwate
thwathe
thwave
thwaze
thweeb
thweech
thweed
thweef
thweeg
thweege
thweek
thweel
thweem
thween
thweep

thweer
thweese
thweesh
thweet
thweeth
thweeve
thweeze
thwobe
thwoche
thwode
thwofe
thwoge
thwogue
thwoke
thwole
thwome
thwone
thwoob
thwooch
thwood
thwoof
thwoog
thwooge
thwook
thwool
thwoom
thwoon
thwoop
thwoor
thwoose
thwoosh
thwoot
thwooth
thwoove
thwooze
thwope
thwore
thwose
thwoshe
thwote
thwothe
thwove
thwoze
tofe
toge
togue

toof
toog
tooge
toosh
toove
toshe
tove
trabe
trache
trafe
trage
trague
trake
trame
treeb
treech
treef
treeg
treege
treek
treem
trobe
troche
trofe
troge
trogue
troke
trome
troob
trooch
troof
troog
trooge
trook
troom
twabe
twache
twafe
twage
twague
twale
twame
twape
tware
twase
twashe
twathe
twave
tweeb
tweech
tweef
tweeg
tweege
tweel
tweem
tweep
tweer
tweese
tweesh
tweeth
tweeve
twobe
twoche
twofe
twoge
twogue
twole
twome
twoob
twooch
twoof
twoog
twooge
twool
twoom
twoop
twoor
twoose
twoosh
twooth
twoove
twope
twore
twose
twoshe
twothe
twove
vabe
vade
vafe
vage
vake
vame
vape
vashe
vave
veeb
veed
veef
veege
veek
veem
veep
veesh
veeve
vobe
vode
vofe
voge
voke
vome
voob
vood
voof
vooge
vook
voom
voop
voosh
voove
vope
voshe
vove
wache
wague
wathe
weech
weeg
weeth
woche
wogue
wooch
woog
wooth
wothe
yabe
yache
yafe
yage
yague
yame
yane
yape
yase
yashe
yate
yave
yaze
yeeb
yeech
yeef
yeeg
yeege
yeem
yeen
yeep
yeese
yeesh
yeet
yeeve
yeeze
yobe
yoche
yofe
yoge
yogue
yome
yone
yoob
yooch
yoof
yoog
yooge
yoom
yoon
yoop
yoose
yoosh
yoot
yoove
yooze
yope
yose
yoshe
yote
yove
yoze
zabe
zache
zade
zafe
zage
zague
zake
zape
zare
zashe
zate
zathe
zave
zeeb
zeech
zeed
zeef
zeeg
zeege
zeek
zeep
zeer
zeesh
zeet
zeeth
zeeve
zobe
zoche
zode
zofe
zoge
zogue
zoke
zoob
zooch
zood
zoof
zoog
zooge
zook
zoop
zoor
zoosh
zoot
zooth
zoove
zope
zore
zoshe
zote
zothe
zove
